# Identification of Novel Genetic Loci Associated with Thyroid Peroxidase Antibodies and Clinical Thyroid Disease

**DOI:** 10.1371/journal.pgen.1004123

**Published:** 2014-02-27

**Authors:** Marco Medici, Eleonora Porcu, Giorgio Pistis, Alexander Teumer, Suzanne J. Brown, Richard A. Jensen, Rajesh Rawal, Greet L. Roef, Theo S. Plantinga, Sita H. Vermeulen, Jari Lahti, Matthew J. Simmonds, Lise Lotte N. Husemoen, Rachel M. Freathy, Beverley M. Shields, Diana Pietzner, Rebecca Nagy, Linda Broer, Layal Chaker, Tim I. M. Korevaar, Maria Grazia Plia, Cinzia Sala, Uwe Völker, J. Brent Richards, Fred C. Sweep, Christian Gieger, Tanguy Corre, Eero Kajantie, Betina Thuesen, Youri E. Taes, W. Edward Visser, Andrew T. Hattersley, Jürgen Kratzsch, Alexander Hamilton, Wei Li, Georg Homuth, Monia Lobina, Stefano Mariotti, Nicole Soranzo, Massimiliano Cocca, Matthias Nauck, Christin Spielhagen, Alec Ross, Alice Arnold, Martijn van de Bunt, Sandya Liyanarachchi, Margit Heier, Hans Jörgen Grabe, Corrado Masciullo, Tessel E. Galesloot, Ee M. Lim, Eva Reischl, Peter J. Leedman, Sandra Lai, Alessandro Delitala, Alexandra P. Bremner, David I. W. Philips, John P. Beilby, Antonella Mulas, Matteo Vocale, Goncalo Abecasis, Tom Forsen, Alan James, Elisabeth Widen, Jennie Hui, Holger Prokisch, Ernst E. Rietzschel, Aarno Palotie, Peter Feddema, Stephen J. Fletcher, Katharina Schramm, Jerome I. Rotter, Alexander Kluttig, Dörte Radke, Michela Traglia, Gabriela L. Surdulescu, Huiling He, Jayne A. Franklyn, Daniel Tiller, Bijay Vaidya, Tim de Meyer, Torben Jørgensen, Johan G. Eriksson, Peter C. O'Leary, Eric Wichmann, Ad R. Hermus, Bruce M. Psaty, Till Ittermann, Albert Hofman, Emanuele Bosi, David Schlessinger, Henri Wallaschofski, Nicola Pirastu, Yurii S. Aulchenko, Albert de la Chapelle, Romana T. Netea-Maier, Stephen C. L. Gough, Henriette Meyer zu Schwabedissen, Timothy M. Frayling, Jean-Marc Kaufman, Allan Linneberg, Katri Räikkönen, Johannes W. A. Smit, Lambertus A. Kiemeney, Fernando Rivadeneira, André G. Uitterlinden, John P. Walsh, Christa Meisinger, Martin den Heijer, Theo J. Visser, Timothy D. Spector, Scott G. Wilson, Henry Völzke, Anne Cappola, Daniela Toniolo, Serena Sanna, Silvia Naitza, Robin P. Peeters

**Affiliations:** 1 Department of Internal Medicine, Erasmus Medical Center Rotterdam, Rotterdam, The Netherlands; 2 Istituto di Ricerca Genetica e Biomedica (IRGB), Consiglio Nazionale delle Ricerche, c/o Cittadella Universitaria di Monserrato, Monserrato, Cagliari, Italy; 3 Dipartimento di Scienze Biomediche, Universita di Sassari, Sassari, Italy; 4 Division of Genetics and Cell Biology, San Raffaele Scientific Institute, Milan, Italy; 5 Interfaculty Institute for Genetics and Functional Genomics, University Medicine and Ernst-Moritz-Arndt-University Greifswald, Greifswald, Germany; 6 Department of Endocrinology and Diabetes, Sir Charles Gairdner Hospital, Nedlands, Western Australia, Australia; 7 Cardiovascular Health Research Unit, Departments of Medicine, Epidemiology and Health Services, University of Washington, Seattle, Washington, United States of America; 8 Institute for Genetic Epidemiology, Helmholtz Zentrum Munich, Munich/Neuherberg, Germany; 9 Department of Endocrinology and Internal Medicine, University Hospital Ghent and Faculty of Medicine, Ghent University, Ghent, Belgium; 10 Internal Medicine, Division of Endocrinology, Radboud University Nijmegen Medical Center, Nijmegen, The Netherlands; 11 Department for Health Evidence, Radboud University Medical Centre, Nijmegen, The Netherlands; 12 Institute of Behavioural Sciences, University of Helsinki, Helsinki, Finland; 13 Oxford Centre for Diabetes, Endocrinology and Metabolism and NIHR Oxford Biomedical Research Centre, Oxford, UK Churchill Hospital, Headington, Oxford, United Kingdom; 14 Research Centre for Prevention and Health, Glostrup University Hospital, the Capital Region of Denmark, Glostrup, Denmark; 15 Genetics of Complex Traits, University of Exeter Medical School, University of Exeter, Exeter, United Kingdom; 16 Peninsula NIHR Clinical Research Facility, University of Exeter Medical School, University of Exeter, Exeter, United Kingdom; 17 Institute of Medical Epidemiology, Biostatistics, and Informatics, Martin-Luther-University Halle-Wittenberg, Halle, Germany; 18 Comprehensive Cancer Center, Ohio State University, Columbus, Ohio, United States of America; 19 Department of Epidemiology, Erasmus Medical Center Rotterdam, Rotterdam, The Netherlands; 20 Departments of Medicine, Human Genetics, Epidemiology and Biostatistics, Lady Davis Institute, McGill University, Montreal, Canada; 21 Department of Twin Research and Genetic Epidemiology, King's College London, London, United Kingdom; 22 National Institute for Health and Welfare, Helsinki, Finland; 23 Hospital for Children and Adolescents, Helsinki University Central Hospital and University of Helsinki, Helsinki, Finland; 24 Institute of Laboratory Medicine, Clinical Chemistry and Molecular Diagnostics, University Hospital Leipzig, Leipzig, Germany; 25 Wellcome Trust Sanger Institute, Hixton, United Kingdom; 26 Institute of Clinical Chemistry and Laboratory Medicine, University Medicine Greifswald, Greifswald, Germany; 27 Department of Biostatistics, University of Washington, Seattle, Washington, United States of America; 28 Helmholtz Zentrum Muenchen, German Research Center for Environmental Health, Institute of Epidemiology II, Neuherberg, Germany; 29 Department of Psychiatry and Psychotherapy, University Medicine Greifswald, HELIOS Hospital Stralsund, Greifswald, Germany; 30 Pathwest Laboratory Medicine WA, Nedlands, Western Australia, Australia; 31 Research Unit of Molecular Epidemiology Helmholtz Zentrum München - German Research Center for Environmental Health, Neuherberg, Germany; 32 School of Medicine and Pharmacology, the University of Western Australia, Crawley, Western Australia, Australia; 33 UWA Centre for Medical Research, Western Australian Institute for Medical Research, Perth, Western Australia, Australia; 34 School of Population Health, University of Western Australia, Nedlands, Western Australia, Australia; 35 MRC Lifecourse Epidemiology Unit, Southampton General Hospital, Southampton, United Kingdom; 36 School of Pathology and Laboratory Medicine, University of Western Australia, Crawley, Western Australia, Australia; 37 High Performance Computing and Network, CRS4, Parco Tecnologico della Sardegna, Pula, Italy; 38 Center for Statistical Genetics, Department of Biostatistics, University of Michigan, Ann Arbor, Michigan, United States of America; 39 Department of Chronic Disease Prevention, National Institute for Health and Welfare, Helsinki, Finland; 40 Vaasa Health Care Centre, Diabetes Unit, Vaasa, Finland; 41 Department of Respiratory Medicine, Sir Charles Gairdner Hospital, Nedlands, Western Australia, Australia; 42 Institute for Molecular Medicine Finland (FIMM), University of Helsinki, Helsinki, Finland; 43 Institute of Human Genetics, Helmholtz Zentrum Munich, Munich, Germany; 44 Institute of Human Genetics, Technische Universität München, Munich, Germany; 45 Department of Cardiology and Internal Medicine, University Hospital Ghent and Faculty of Medicine, Ghent University, Ghent, Belgium; 46 Wellcome Trust Sanger Institute, Wellcome Trust Genome Campus, Cambridge, United Kingdom; 47 Department of Medical Genetics, University of Helsinki and University Central Hospital, Helsinki, Finland; 48 Diagnostica Stago, Doncaster, Victoria, Australia; 49 Institute for Translational Genomics and Population Sciences, Los Angeles Biomedical Research Institute, Torrance, California, United States of America; 50 Department of Pediatrics, Harbor-UCLA Medical Center, Torrance, California, United States of America; 51 Institute for Community Medicine, University Medicine Greifswald, Greifswald, Germany; 52 Department of Twin Research and Genetic Epidemiology, King's College London, London, United Kingdom; 53 School of Clinical and Experimental Medicine, College of Medical and Dental Sciences, Univeristy of Birmingham, Edgbaston, Birmingham, United Kingdom; 54 Diabetes, Endocrinology and Vascular Health Centre, Royal Devon and Exeter NHS Foundation Trust, Exeter, United Kingdom; 55 BIOBIX Lab. for Bioinformatics and Computational Genomics, Dept. of Mathematical Modelling, Statistics and Bioinformatics. Faculty of Bioscience Engineering, Ghent University, Ghent, Belgium; 56 Faculty of Health Science, University of Copenhagen, Copenhagen, Denmark; 57 Department of General Practice and Primary Health Care, University of Helsinki, Helsinki, Finland; 58 Helsinki University Central Hospital, Unit of General Practice, Helsinki, Finland; 59 Folkhalsan Research Centre, Helsinki, Finland; 60 Vasa Central Hospital, Vasa, Finland; 61 Curtin Health Innovation Research Institute, Curtin University of Technology, Bentley, Western Australia, Australia; 62 Institute of Epidemiology I, Helmholtz Zentrum Munich, Munich, Germany; 63 Group Health Research Institute, Group Health Cooperative, Seattle, Washington, United States of America; 64 Department of Internal Medicine, Diabetes & Endocrinology Unit, San Raffaele Scientific Institute and Vita-Salute San Raffaele University, Milan, Italy; 65 Laboratory of Genetics, National Institute on Aging, Baltimore, Maryland, United States of America; 66 Institute for Maternal and Child Health - IRCCS “Burlo Garofolo”, Trieste, Italy; 67 University of Trieste, Trieste, Italy; 68 Biopharmacy, Department of Pharmaceutical Sciences, University Basel, Basel, Switzerland; 69 Netherlands Consortium for Healthy Aging, Netherlands Genomics Initiative, Leiden, The Netherlands; 70 Department of Internal Medicine, VU Medical Center, Amsterdam, The Netherlands; 71 Division of Endocrinology, Diabetes, and Metabolism, Perelman School of Medicine at the University of Pennsylvania, Philadelphia, Pennsylvania, United States of America; 72 Institute of Molecular Genetics-CNR, Pavia, Italy; Yale School of Medicine, United States of America

## Abstract

Autoimmune thyroid diseases (AITD) are common, affecting 2-5% of the general population. Individuals with positive thyroid peroxidase antibodies (TPOAbs) have an increased risk of autoimmune hypothyroidism (Hashimoto's thyroiditis), as well as autoimmune hyperthyroidism (Graves' disease). As the possible causative genes of TPOAbs and AITD remain largely unknown, we performed GWAS meta-analyses in 18,297 individuals for TPOAb-positivity (1769 TPOAb-positives and 16,528 TPOAb-negatives) and in 12,353 individuals for TPOAb serum levels, with replication in 8,990 individuals. Significant associations (*P*<5×10^−8^) were detected at *TPO*-rs11675434, *ATXN2*-rs653178, and *BACH2-*rs10944479 for TPOAb-positivity, and at *TPO-*rs11675434, *MAGI3*-rs1230666, and *KALRN*-rs2010099 for TPOAb levels. Individual and combined effects (genetic risk scores) of these variants on (subclinical) hypo- and hyperthyroidism, goiter and thyroid cancer were studied. Individuals with a high genetic risk score had, besides an increased risk of TPOAb-positivity (OR: 2.18, 95% CI 1.68–2.81, *P* = 8.1×10^−8^), a higher risk of increased thyroid-stimulating hormone levels (OR: 1.51, 95% CI 1.26–1.82, *P* = 2.9×10^−6^), as well as a decreased risk of goiter (OR: 0.77, 95% CI 0.66–0.89, *P* = 6.5×10^−4^). The *MAGI3* and *BACH2* variants were associated with an increased risk of hyperthyroidism, which was replicated in an independent cohort of patients with Graves' disease (OR: 1.37, 95% CI 1.22–1.54, *P* = 1.2×10^−7^ and OR: 1.25, 95% CI 1.12–1.39, *P* = 6.2×10^−5^). The *MAGI3* variant was also associated with an increased risk of hypothyroidism (OR: 1.57, 95% CI 1.18–2.10, *P* = 1.9×10^−3^). This first GWAS meta-analysis for TPOAbs identified five newly associated loci, three of which were also associated with clinical thyroid disease. With these markers we identified a large subgroup in the general population with a substantially increased risk of TPOAbs. The results provide insight into why individuals with thyroid autoimmunity do or do not eventually develop thyroid disease, and these markers may therefore predict which TPOAb-positives are particularly at risk of developing clinical thyroid dysfunction.

## Author Summary

Individuals with thyroid peroxidase antibodies (TPOAbs) have an increased risk of autoimmune thyroid diseases (AITD), which are common in the general population and associated with increased cardiovascular, metabolic and psychiatric morbidity and mortality. As the causative genes of TPOAbs and AITD remain largely unknown, we performed a genome-wide scan for TPOAbs in 18,297 individuals, with replication in 8,990 individuals. Significant associations were detected with variants at *TPO*, *ATXN2*, *BACH2*, *MAGI3*, and *KALRN*. Individuals carrying multiple risk variants also had a higher risk of increased thyroid-stimulating hormone levels (including subclinical and overt hypothyroidism), and a decreased risk of goiter. The *MAGI3* and *BACH2* variants were associated with an increased risk of hyperthyroidism, and the *MAGI3* variant was also associated with an increased risk of hypothyroidism. This first genome-wide scan for TPOAbs identified five newly associated loci, three of which were also associated with clinical thyroid disease. With these markers we identified a large subgroup in the general population with a substantially increased risk of TPOAbs. These results provide insight into why individuals with thyroid autoimmunity do or do not eventually develop thyroid disease, and these markers may therefore predict which individuals are particularly at risk of developing clinical thyroid dysfunction.

## Introduction

Autoimmune thyroid disease (AITD), including Hashimoto's thyroiditis and Graves' disease, is one of the most common autoimmune diseases, affecting 2–5% of the general population [1], [2], [3]. Thyroid dysfunction has been associated with osteoporosis, depression, atrial fibrillation, heart failure, metabolic syndrome, and mortality [4], [5], [6], [7], [8], [9], [10], [11]. High serum antibodies against the enzyme thyroid peroxidase (TPO), which is located in the thyroid and plays a key role in thyroid hormone synthesis, are present in 90% of patients with Hashimoto's thyroiditis [12], [13], the most frequent cause of hypothyroidism and goiter. Although TPO antibodies (TPOAbs) are a useful clinical marker for the detection of early AITD, it remains controversial if these antibodies play a causative role in the pathogenesis of Hashimoto's thyroiditis [14], [15], [16].

Interestingly, TPOAb-positive persons also have an increased risk of developing autoimmune hyperthyroidism (Graves' disease) [17], [18], which is caused by stimulating antibodies against the thyroid stimulating hormone (TSH) receptor [19]. Numerous studies have shown that Graves' hyperthyroidism and Hashimoto's thyroiditis show co-inheritance [17], [20], [21]. Finally, thyroid autoimmunity is the most common autoimmune disorder in women of childbearing age, and TPOAb-positive women have an increased risk of developing pregnancy complications such as miscarriage and pre-term delivery [17], [18], [22], [23], [24], [25], [26].

The prevalence of TPOAb-positivity in the general population ranges from 5–24%, but it is currently unknown why these people develop TPOAbs, nor is it known why not all individuals with thyroid autoimmunity develop clinical thyroid disease [27], [28]. It is estimated that around 70% of the susceptibility to develop thyroid autoantibodies is due to genetic factors [29]. In this context it is remarkable to note that little is known about the genetic factors that determine TPOAb-positivity and the risk of AITD.

We therefore performed a genome wide association study (GWAS) meta-analysis for TPOAbs in the general population in 18,297 individuals from 11 populations. Newly identified genetic variants were studied in relation to subclinical and overt hypo- and hyperthyroidism, goiter, thyroid autoimmunity during pregnancy and thyroid cancer risk.

## Results

Characteristics of the studied populations are shown in Table 1 and the Supplementary Material S1. Heritability estimates in the family-based cohorts SardiNIA, TwinsUK and Val Borbera were, respectively, 0.65, 0.66, and 0.54 for TPOAb-positivity, and 0.43, 0.66, and 0.30 for TPOAb levels.

**Table 1:** Population characteristics and serum TPOAb, TSH, and FT4 level measurements specifications.

		Sample characteristics						TPOAb specifications			TSH specifications		FT4 specifications	
Study	Ethnic group (origin)	N with TPOAb and GWAS data	N using thyroid medication	N case-control approach (cases/controls)	N continuous approach	Men (%)	Age (yrs) Mean (SD)	TPOAb-positivity (%)	TPOAb-positivity cut off	Assay (Detection range)	TSH Median (IQR)	Assay (normal range)	FT4 Mean (SD)	Assay (normal range)
Stage 1														
BHS	Caucasian (Australia)	1363	47	1316 (197/1119)	1316	43%	53.0 (17.2)	15.0%	35	Immulite 2000 chemiluminescent immunoassay (5-5000)	1.3 (0.9;1.9) mU/L	Immulite 2000 chemiluminescent immunoassay (0.4 - 4.0 mU/L)	16.9 (2.5) pmol/L	Immulite 2000 chemiluminescent immunoassay (9 – 23 pmol/L)
CHS	Caucasian (USA)	2024	0	2024 (281/1743)	1817	41%	74.8 (5.1)	13.9%	34	Chemiluminescent immunoassay (5–600)	2.3 (1.5;3.5) mU/L	Chemiluminescent immunoassay (0.27–4.2 mU/L)	1.2 (0.2) ng/dL	Chemiluminescent immunoassay (0.93–1.7 ng/dL)
HBCS	Caucasian (Finland)	526	29	497 (75/422)	497	50%	61.0 (2.8)	15.1%	12	Chemiluminescent immunoassay (0–1000)	2.0 (1.2;2.4) mU/L	Chemiluminescent immunoassay (0.49–4.67 mU/L)	14.1 (1.6) ng/dL	Chemiluminescent immunoassay (0.71–1.85 ng/dL)
KORA	Caucasian (Germany)	1765	49	1475 (74/1401)	1475	45%	60.5 (8.9)	5.0%	200	Chemiluminescent immunoassay (1–3000)	1.5 (0.6;2.5) mU/L	Chemiluminescent immunoassay (0.4–4.3 mU/L)	18.9 (2.6) pmol/L	Chemiluminescent immunoassay (11–25 pmol/L)
NBS	Caucasian (Netherlands)	1829	26	1829 (287/1542)	1829	50%	61.5 (10.3)	15.7%	12	Fluoro-immunometric assay (2.6–1000)	1.3 (0.9;2.0) mU/L	Immuno-luminometric assay (0.4–4.0 mU/L)	13.5 (2.4) pmol/L	Chemiluminescent immunoassay (8.0–22.0 pmol/L)
RS	Caucasian (Netherlands)	1627	50	1577 (137/1440)	210	40%	70.2 (5.6)	8.7%	35	Chemiluminescent immunoassay (5–5000)	1.2 (0.6:2.5) mU/L	Chemiluminescent immunoassay (0.4–4.3 mU/L)	18.4 (2.4) pmol/L	Chemiluminescent immunoassay (11–25 pmol/L)
SardiNIA	Caucasian (Italy)	4686	154	972 (108/864)	1257	49%	56.9 (12.5)	11.1%	35	Chemiluminescent immunoassay (5–1000)	1.3 (0.8:2.0)mU/L	Chemiluminescent immunoassay (0.4–4.0 mU/L)	1.3 (0.2) ng/dL	Chemiluminescent immunoassay (0.3–2.4 ng/dl)
SHIP	Caucasian (Germany)	4096	293	3803 (265/3538)	1818	52%	49.3 (16.3)	7.0%	60	Chemiluminescent immunoassay (1–3000)	0.7 (0.4;1.0) mU/L	Chemiluminescent immunoassay (0.3–3.0 mU/L)	12.8 (3.8) pmol/L	Chemiluminescent immunoassay (7.7– 23.2 pmol/L)
SHIP-Trend	Caucasian (Germany)	986	99	887 (36/851)	887	46%	49.5 (13.7)	4.1%	200	Chemiluminescent immunoassay (1–3000)	1.2 (0.8;1.6) mU/L	Chemiluminescent immunoassay (0.36–3.74 mU/L)	-	-
TwinsUK	Caucasian (UK)	2455	86	2369 (461/1893)	774	0%	46.9 (12.5)	19.5%	6	Chemiluminescent immunoassay (0.5–1000)	1.3 (0.9;1.8) mU/L	Chemiluminescent immunoassay0.4–4.0 mU/L)	13.6 (1.9) pmol/L	Chemiluminescent immunoassay (9–19 pmol/L)
ValBorbera	Caucasian (Italy)	1661	90	1571 (161/1410)	452	46%	54.3 (18.4)	10.2%	60 and 50	Two chemiluminescent immunoassays (5.5–3000 ; 6-7500)	1.4 (0.9;2.0) mU/L	Chemiluminescent immunoassay (0.34–5.60 mU/L)	-	-

		Sample characteristics						TPOAb specifications			TSH specifications		FT4 specifications	
Study	Ethnic group (origin)	N with TPOAb and GWAS data	N using thyroid medication	N case-control approach (cases/controls)	N continuous approach	Men (%)	Age (yrs) Mean (SD)	TPOAb-positivity (%)	TPOAb-positivity cut-off	Assay (Detection range)	TSH Median (IQR)	Assay (normal range)	FT4 Mean (SD)	Assay (normal range)
Stage 2														
Asklepios	Caucasian (Belgium)	2418	109	2309 (245/2064)	2185	50%	45.9 (5.9)	10.6%	35	Chemiluminescent immunoassay (5–600)	1.5 (1.1;2.1) mU/L	Chemiluminescent immunoassay (0.3–4.2 mU/L)	1.31 (0.2) ng/dL	Chemiluminescent immunoassay (0.9–1.7 ng/dl)
CARLA	Caucasian (Germany)	1753	270	1483 (186/1297)	1190	60%	64.2 (10.2)	12.5%	28	Chemiluminescent immunoassay (5–600)	0.9 (0.6;1.2) mU/L	Chemiluminescent immunoassay (0.4– 3.8 mU/L)	16.3 (2.5) pmol/L	Chemiluminescent immunoassay (12.8–20.4 pmol/L)
EFSOCH	Caucasian (UK)	1289	-	1289 (97/1192)	1233	64%	34.2 (5.9)	7.5%	34	Chemiluminescent immunoassay (5–600)	1.9 (1.3;2.6) mU/L	Chemiluminescent immunoassay (0.4–4.5 mU/L)	16.0 (2.4) pmol/L	Chemiluminescent immunoassay (11–24 pmol/L)
Health2006 Study	Caucasian (Danish)	3287	-	3287 (204/3083)	3285	45%	49.3 (13.0)	6.2%	100	Chemiluminescent immunoassay (1–3000)	1.7 (1.0;2.0) mU/L	Chemiluminescent immunoassay (0.4–3.7 mIU/L)	12.4 (1.8) pmol/L	Chemiluminescent immunoassay (9.8–18.8 pmol/L)
SardiNIA2	Caucasian (Italy)	1387	30	765 (104/661)	375	41%	46.6 (17.4)	13.6%	35	Chemiluminescent immunoassay (5–1000)	1.6 (1.0;2.2) mIU/ml	Chemiluminescent immunoassay (0.4– 4.0 mU/L)	1.3 (0.2) ng/dl	Chemiluminescent immunoassay (0.3–2.4 ng/dl)

### Loci associated with TPOAb-positivity and TPOAb levels

See Table 1 and Supplementary Figure S1 for TPOAb measurements and Supplementary Table S1 for genotyping procedures. In most autoimmune diseases, both the presence and the level of autoantibodies are relevant for the disease onset [18], [30], [31]. Furthermore, different pathophysiological processes may be involved in the initiation and severity of the autoimmune response. We therefore performed a GWAS on TPOAb-positivity (including 1769 TPOAb-positives and 16,528 TPOAb–negatives), as well as a GWAS on continuous TPOAb levels (including 12,353 individuals) in stage 1. See Supplementary Figures S2 and S3 for QQ (quantile-quantile) and Manhattan plots.

**Figure 1:**
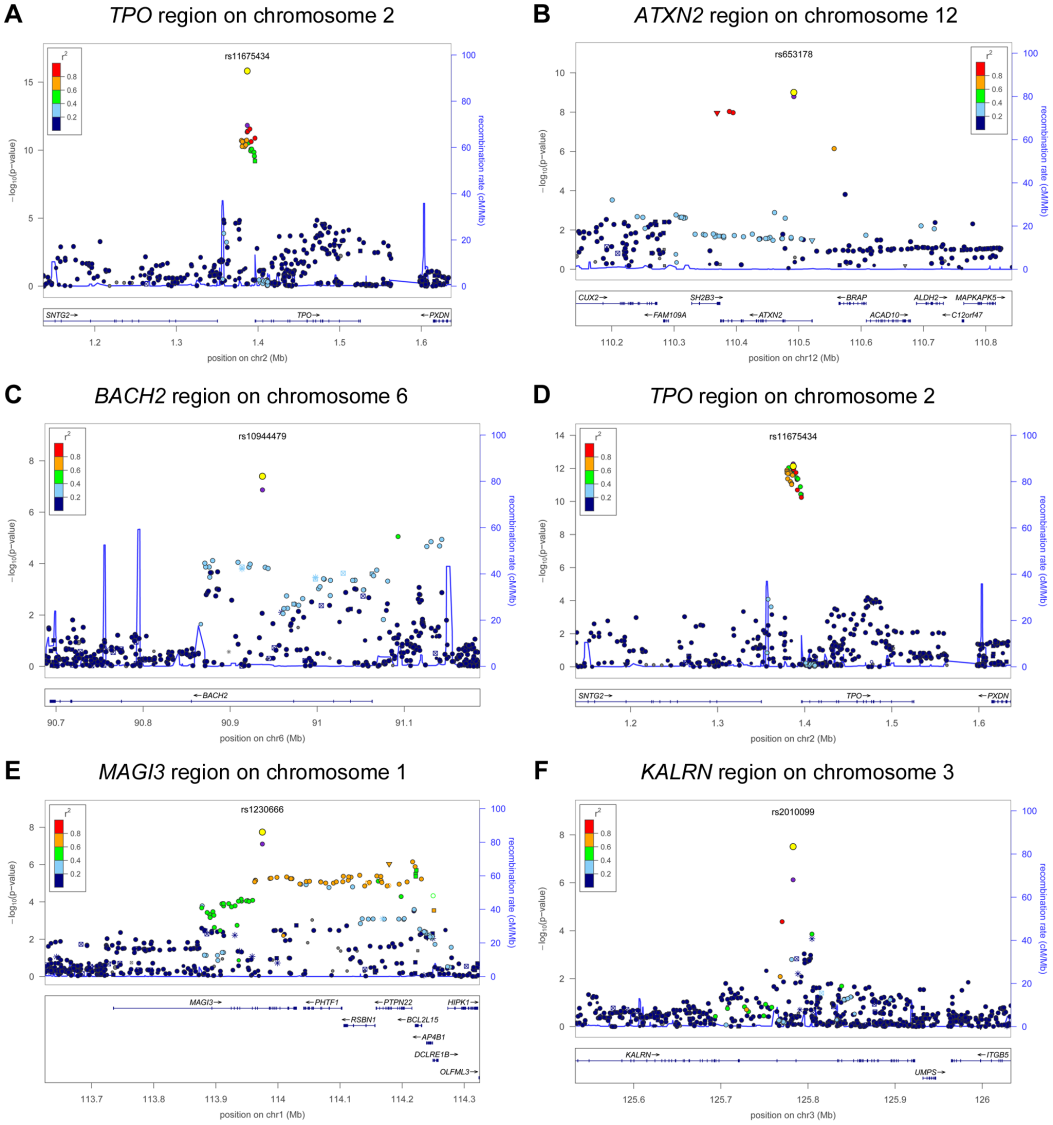
Genome wide association studies meta-analyses: Loci associated with TPOAb-positivity (a–c) and TPOAb levels (d–f) on a genome-wide level of significance. Regional association plots of the genome-wide significant loci associated with TPOAb positivity (a–c) and TPOAb levels (d–f). The y-axis on the left indicates the – log_10_ *P* value for the association with TPOAb –positivity (a–c) or TPOAb levels (d–f). SNPs are plotted on the x-axis according to their chromosomal position against the association with the phenotype on the y-axis. The most significant stage 1 SNP is indicated in purple. The combined stage 1 and 2 result of this SNP is indicated in yellow. The SNPs surrounding the most significant SNP are color-coded to reflect their LD with this SNP. Symbols reflect functional genomic annotation, as indicated in the legend. The blue y-axes on the right of each plot indicate the estimated recombination rates (based on HapMap Phase II); the bottom of each panel shows the respective annotated genes at the locus and their transcriptional direction. Mb, megabases.

In stage 2, we followed-up 20 stage 1 SNPs (*P*<5×10^−6^; 13 TPOAb-positivity and 10 TPOAb level SNPs, with 3 SNPs overlapping) in 5 populations, including up to 8,990 individuals for TPOAb-positivity (922 TPOAb-positives and 8068 TPOAb–negatives) and 8,159 individuals for TPOAb level analyses (see Supplementary Material S1). Results of the combined stage 1 and 2 meta-analyses, including heterogeneity analyses, are shown in Supplementary Tables S2 and S3. Regional association plots are shown in Supplementary Figures S4 and S5. In the combined stage 1 and 2 meta-analyses GWAS significant associations (*P*<5×10^−8^) were observed near *TPO* (Chr 2p25; rs11675434), at *ATXN2* (Chr 12q24.1; rs653178), and *BACH2* (Chr 6q15; rs10944479) for TPOAb-positivity, and near *TPO* (rs11675434), at *MAGI3* (Chr 6q15; rs1230666), and *KALRN* (Chr 3q21; rs2010099) for TPOAb levels (Table 2 and Figure 1). The TPOAb level meta-analysis *P*-values for the 3 GWAS significant TPOAb-positivity loci were: *TPO*-rs11675434: *P* = 7.4×10^−13, *ATXN2*^-rs653178: *P* = 1.3×10^−7, and^ *BACH2-*rs10944479: *P* = 2.0×10^−4.^

**Table 2:** Newly identified loci associated with TPOAb-positivity and/or serum TPOAb levels reaching genome wide significance.

				Alleles				Stage 1 + 2 meta-analysis: up to 2691 cases and 24,596 controls	
*TPOAb-positivity*	SNP	Chr.	Position (Build 36)	Risk	Other	RAF^a^	Nearby gene	OR (95% CI)^b^	*P* value
	rs11675434	2	1386822	T	C	0.39	*TPO*	1.21 (1.15–1.28)	1.5×10^−16^
	rs653178	12	110492139	C	T	0.40	*ATXN2*	1.14 (1.08–1.19)	9.9×10^−10^
	rs10944479	6	90937114	A	G	0.16	*BACH2*	1.25 (1.14–1.37)	4.0×10^−8^
				Alleles				Stage 1 + 2 meta-analysis: up to 20,512 subjects	
*TPOAb levels*	SNP	Chr.	Position (Build 36)	Risk	Other	RAF^a^	Nearby gene	*β* (SE)^c^	*P* value
	rs11675434	2	1386822	T	C	0.39	*TPO*	0.0202 (0.0046)	7.4×10^−13^
	rs1230666	1	113974933	A	G	0.16	*MAGI3*	0.0269 (0.0064)	1.8×10^−8^
	rs2010099	3	125782947	C	T	0.91	*KALRN*	0.0240 (0.0076)	3.1×10^−8^

Chr., chromosome

a Risk allele frequency: Weighted mean frequency of the risk allele across all included cohorts.

b Adjusted for age and gender

c Expressed in sd of natural logarithm transformed serum TPOAb level, adjusted for age and gender

As the 3 GWAS significant loci for TPOAb levels also showed associations with TPOAb-positivity (*TPO*-rs11675434*:* OR, 1.21 [95% CI, 1.15–1.28)], *P* = 1.5×10^−16^; *MAGI3*-rs1230666*:* OR, 1.23 [95% CI, 1.14–1.33], *P* = 1.5×10^−6^; *KALRN*-rs2010099*:* OR, 1.24 [95% CI, 1.12–1.37], *P* = 7.4×10^−5^), we subsequently studied the (combined) effects of these 5 SNPs on clinical thyroid disease. Genetic risk scores were calculated as described in the Supplementary Material. The variance explained by these 5 SNPs was 3.1% for TPOAb-positivity and 3.2% for TPOAb levels. Subjects with a high genetic risk score had a 2.2 times increased risk of TPOAb-positivity compared to subjects with a low genetic risk score (*P* = 8.1×10^−8^) (Table 3).

**Table 3:** Genetic risk score and the risk of TPOAb-positivity.

GRS Quartile	% TPOAb-positivity (N cases/total)	OR (95% CI)^a^	*P* value
1 (reference)	5.4% (89/1637)	-	-
2	7.0% (114/1637)	1.29 (0.98–1.69)	0.07
3	9.0% (152/1695)	1.64 (1.26–2.13)	1.3×10^−4^
4	10.4% (158/1523)	2.18 (1.68–2.81)	8.1×10^−8^

GRS, genetic risk score (based on rs11675434, rs653178, rs10944479, rs1230666, rs2010099).

a Adjusted for age and gender

Table S4 shows the stage 1 TPOAb-positivity and TPOAb level meta-analyses results for GWAS significant SNPs reported in previous GWAS on thyroid related phenotypes.

### Associations with hypo- and hyperthyroidism

The associations between the 5 GWAS significant SNPs and the risk of abnormal thyroid function tests are shown in Table 4. *MAGI3*- rs1230666 was associated with an increased risk of overt hypothyroidism and increased TSH levels below the Bonferroni threshold (i.e., *P = *0.05/5 = 0.01). Borderline significant signals were observed at *BACH2*- rs10944479 with a higher risk of increased TSH levels as well as overt hyperthyroidism (*P = *0.011 and *P = *0.012), and at the *KALRN*-rs2010099 SNP with a lower risk of decreased TSH levels (*P = *0.010).

**Table 4:** Newly identified TPOAb associated loci and the risk of thyroid disease in stage 1 and 2 populations.

		Alleles		Increased TSH (1110 cases/19,189 controls)		Hypothyroidism (173 cases/15,940 controls)		Decreased TSH (967 cases/19,297 controls)		Hyperthyroidism (78 cases/14,901 controls)	
Nearby gene	SNP	Risk	Other	OR (95% CI)	*P* value	OR (95% CI)^a^	*P* value	OR (95% CI)^a^	*P* value	OR (95% CI)^a^	*P* value
*TPO*	rs11675434	T	C	1.08 (0.99–1.18)	0.08	1.14 (0.91–1.42)	0.26	1.02 (0.93–1.11)	0.68	1.10 (0.81–1.49)	0.54
*ATXN2*	rs653178	C	T	1.01 (0.98–1.04)	0.68	1.25 (1.01–1.54)	0.04	1.01 (0.97–1.04)	0.70	1.00 (0.74–1.33)	0.99
*BACH2*	rs10944479	A	G	1.17 (1.04–1.32)	0.011	1.37 (1.00–1.88)	0.05	0.91 (0.80–1.03)	0.15	1.80 (1.14–2.85)	0.012
*MAGI3*	rs1230666	A	G	1.23 (1.09–1.39)	9.0×10^−4^	1.57 (1.18–2.10)	1.9×10^−3^	1.08 (0.96–1.22)	0.22	1.61 (0.99–2.60)	0.05
*KALRN*	rs2010099	C	T	1.05 (0.90–1.23)	0.52	0.80 (0.54–1.20)	0.28	0.82(0.71–0.95)	0.010	0.69 (0.39–1.24)	0.21

All analyses adjusted for age and gender.

*ATXN2*-rs653178 is in high LD with *SH2B3-* rs3184504

*MAGI3-*rs1230666 is in high LD with *PTPN22-*rs2476601

Furthermore, a higher genetic risk score was associated with a higher risk of increased TSH levels (Supplementary Table S5). No effects of the genetic risk score on the risk of overt hypothyroidism, hyperthyroidism or decreased TSH levels were observed.

### Associations with goiter

Individuals with a high genetic risk score had a 30.4% risk of sonographically-proven goiter, compared to 35.2% in subjects with a low score (*P* = 6.5×10^−4^) (Table 5). None of the individual SNPs was significantly associated with goiter risk.

**Table 5:** Newly identified TPOAb associated loci, genetic risk scores and the risk of goiter.

*TPO*	rs11675434	T	C	0.95 (0.88–1.02)	0.17	1 (reference)	35.2% (588/1669)	-	-
*ATXN2*	rs653178	C	T	0.95 (0.88–1.03)	0.22	2	33.7% (570/1691)	0.92 (0.79–1.06)	0.21
*BACH2*	rs10944479	A	G	0.94 (0.85–1.05)	0.28	3	31.6% (530/1675)	0.84 (0.72–0.98)	0.03
*MAGI3*	rs1230666	A	G	0.90 (0.81–1.00)	0.05	4	30.4% (517/1702)	0.77 (0.66–0.89)	6.5×10^−4^
*KALRN*	rs2010099	C	T	0.93 (0.81–1.05)	0.23				

GRS, genetic risk score (based on rs11675434, rs653178, rs10944479, rs1230666, rs2010099).

a Adjusted for age, gender, and body surface area.

*ATXN2*-rs653178 is in high LD with *SH2B3*-rs3184504.

*MAGI3*-rs1230666 is in high LD with *PTPN22*-rs24756601.

### Thyroid autoimmunity during pregnancy

As autoimmunity significantly changes during pregnancy [25], we additionally studied these effects in an independent pregnant population. Pregnant women with a high genetic risk score had a 2.4 times increased risk of TPOAb-positivity compared to women with a low score (10.3% vs 4.8%, *P* = 0.03). These women did not have a higher risk of increased TSH levels. However, a borderline significant signal with a lower risk of increased TSH levels was observed at *ATXN2*- rs653178 (OR, 0.54 [95% CI, 0.34–0.87], *P* = 0.012).

### Associations with thyroid disease in independent populations

#### a) Graves' disease

As *MAGI3*- rs1230666 and *BACH2*- rs10944479 showed promising associations (i.e., *P*≤0.05) with hyperthyroidism in our meta-analyses, we tested these SNPs in an independent population of 2478 patients with Graves' disease and 2682 controls (see Supplementary Material for further details). Both were associated with an increased risk of Graves' disease (*MAGI3*- rs1230666: OR, 1.37 [95% CI, 1.22–1.54]; *P* = 1.2×10^−7;^ *BACH2*- rs10944479: OR, 1.25 [1.12–1.39]; *P* = 6.2×10^−5^).

#### b) Thyroid cancer

Supplementary Table S6 shows the associations of the 5 GWAS significant SNPs with thyroid cancer. No statistically significant associations were detected, but a borderline significant signal with an increased risk of thyroid cancer was observed at *ATXN2*- rs653178 (OR, 1.32 [95% CI, 1.02–1.70], *P* = 0.03).

### Pathway analyses

Ingenuity Pathway Analyses (IPA; Ingenuity Systems, Ca, USA) and GRAIL analyses [32] were performed to identify potential pathways involved in AITD, the results of which are shown in Supplementary Tables S7 and S8, and Figure S6. The identified top pathways involved cell death, survival, movement, and OX40 signalling.

## Discussion

This is the first GWAS meta-analysis investigating the genetics of TPOAbs in the normal population in up to 18,297 individuals from 11 populations with replication in up to 8,990 individuals from 5 populations. We identified 5 GWAS significant loci associated with TPOAb-positivity and/or levels.

The most significant hit for both TPOAb-positivity and TPOAb levels was located near the *TPO* gene itself. TPO is a membrane-bound protein located on the apical membranes of the thyroid follicular cell, catalyzing key reactions in thyroid hormone synthesis [33]. Mutations in *TPO* have been found in patients with congenital hypothyroidism [34], [35]. Although TPOAbs are valid clinical biomarkers of AITD, they are generally considered to be secondary to the thyroid damage inflicted by T-cells.

The *FOXE1* gene has been previously associated with hypothyroidism [36], [37] and is known to regulate transcription of *TPO* [38]. In this context it is interesting to note that we did not find any associations of the variant near *TPO* with hypothyroidism. Most genes that have been associated with AITD (predominantly Graves' disease) by candidate gene and GWAS studies so far are located in the HLA class I and II regions, or in genes involved in T-cell (i.e., *CTLA-4*, *PTPN22*) or other autoimmune responses [28], [39]. Until now, the *TPO* gene itself had not been associated with AITD, except in one recent candidate gene analysis in a small cohort (n = 188) without replication [40]. A variant near *TPO* (rs11694732), which is in LD with rs11675434 (r2 = 0.97 in HapMap2), has previously been associated with TSH levels by Gudmundsson et al [41]. However, various other GWAS on serum TSH and FT4 levels have not found any significant associations in or near this locus, including a recent similar sized GWAS by Porcu et al [42].

Three of the other four loci identified here are located in or are in linkage disequilibrium (LD) with genes previously associated with other autoimmune diseases. Rs1230666 is located in intron 9 of *MAGI3*, encoding a protein that modulates activity of AKT/PKB. AKT/PKB is expressed in the thyroid and regulates apoptosis [43], which seems to play an important role in the development of AITD [44], [45]. In addition, rs1230666 is in LD with rs2476601 (r2 = 0.70 in HapMap2), a variant causing a R620W substitution in *PTPN22*. PTPN22 is a lymphoid-specific intracellular phosphatase involved in the T-cell receptor signaling pathway. Variations in *PTPN22*, and specifically R620W, are associated with various autoimmune disorders including type 1 diabetes, rheumatoid arthritis, systemic lupus erythematosus and Graves' disease [46], [47], [48], [49]. The associations of the *MAGI3* locus with TPOAb-positivity and Graves' disease may therefore also be explained by linkage with disease-associated variants in *PTPN22* [50]. Of note, the association signal at rs2476601 is one order weaker than that of the top variant rs1230666.

The *BACH2* locus has been implicated in the susceptibility to several autoimmune diseases, including celiac disease, type 1 diabetes, vitiligo, Crohn's disease, and multiple sclerosis [46], [51], [52], [53], [54]. A recent candidate gene analysis associated the *BACH2* locus with an increased risk of AITD, including Hashimoto's thyroiditis and Graves' disease [55]. However, the associations were not significant when Hashimoto's thyroiditis and Graves' disease were studied separately. *BACH2* is specifically expressed in early stages of B-cell differentiation and represses different immunoglobulin genes [56]. Interestingly, BACH2 can bind to the co-repressor SMRT (silencing mediator of retinoid and thyroid receptor), which may suggest a more direct effect on thyroid hormone secretion and action as well.

Polymorphisms in *ATXN2* have been associated with multiple neurodegenerative diseases, including spinocerebellar ataxia and Parkinson's disease [57], [58], [59]. Different epidemiological studies have associated thyroid dysfunction with cerebellar ataxia [60], [61]. Furthermore, the identified SNP in *ATXN2* has been previously associated with renal function, serum urate levels and blood pressure [62], [63], [64]. However, this SNP is in high LD with rs3184504 (r2 = 0.873), a variant causing a Trp262Arg substitution of *SH2B adaptor protein 3* (*SH2B3)*. *SH2B3* encodes the adaptor protein LNK, a key negative regulator of cytokine signaling playing a critical role in hematopoiesis. This variant is associated with susceptibility to several autoimmune diseases, including celiac disease, type 1 diabetes, vitiligo, and rheumatoid arthritis [46], [51], [53], [65], suggesting more relevance for TPOAb levels than ATXN2. This is supported by a recent study which showed that variants in LD with *SH2B3*, *BACH2*, and *PTPN22* are associated with TPOAb levels in patients with type 1 diabetes [66].

Whereas the above four loci are located in genes involved in the immune response or the autoantigen, the *KALRN* (*Kalirin*) gene encodes a multi-domain guanine nucleotide exchange factor for GTP-binding proteins of the Rho family. The relation of *KALRN* with levels of TPOAbs is unclear. This gene has recently been found to be associated with megakaryopoiesis and platelet formation [67], which may suggest a function in the immune system [68]. We furthermore performed pathway analyses on the stage 1 TPOAb-positivity and TPOAb level lead SNPs, and identified the cell death, survival and movement pathway as an important pathway for TPOAbs. This finding is supported by previous studies, which show an important role for apoptosis in the development of AITD [44], [45]. Another top pathway involved was the OX40 signalling pathway, and it is of interest to note that OX40 is a T-cell activator promoting the survival of CD4+ T-cells at sites of inflammation [69].

Our results have potential clinical relevance for several reasons. Genetic risk scores based on these novel common (risk allele frequencies: 9–40%) TPOAb-associated SNPs enabled us to identify a large subgroup in the general population with a two-fold increased risk of TPOAb-positivity (10.4% vs 5.4%). These individuals also have a higher risk of increased TSH levels and a lower risk of goiter, suggesting an advanced stage of destruction of the thyroid due to autoimmune processes. Furthermore, pregnant women with high genetic risk scores had a 2.4 times increased risk of TPOAb-positivity during pregnancy. In this context it is interesting to note that TPOAb-positive pregnant women have an increased risk of miscarriages and preterm births independent of thyroid function [70].

Associations with thyroid disease were also found on an individual SNP level. The *MAGI3* SNP was associated with a substantially increased risk of hypothyroidism, and the *BACH2* SNP showed a borderline significant association (*P* = 0.011) with a higher risk of increased TSH levels, which includes subjects with subclinical and overt hypothyroidism. Furthermore, both loci were significantly associated with an increased risk of Graves' hyperthyroidism in an independent population. To predict which patients with first or second degree relatives with documented Hashimoto's or Graves' disease will develop clinical thyroid disease, a clinical algorithm has been developed (i.e., the THEA score) [18]. Future studies should analyze if these genetic markers increase the sensitivity of the THEA score. Graves' hyperthyroidism and Hashimoto's thyroiditis co-segregate in families and subjects with TPOAbs have an increased risk of both diseases [17], [18], [20], [21], [22], [26]. The current study provides insight into this phenomenon by showing that specific loci associated with TPOAbs and (subclinical) hypothyroidism, *i.e. MAGI3* and *BACH2*, are also associated with Graves' hyperthyroidism in an independent case-control study.

The prevalence of TPOAb-positivity in the general population is high (5–24%), but it is currently unknown why part of the individuals with thyroid autoimmunity develop clinical thyroid disease whereas others do not [27], [28]. In this context it is interesting to note that the TPOAb-associated SNPs located in *TPO* and *ATXN2* were not associated with clinical thyroid disease. This suggests that the TPOAbs in these individuals may be of less clinical relevance, providing insight into why TPOAb-positive individuals do or do not eventually develop clinical thyroid disease.

Our study has some limitations. The validity of the results is restricted to individuals from populations of European ancestry. Future GWASs in populations from non-European descent will be required to determine to which extent our results can be generalized to other ethnic groups. Secondly, we did not perform conditional analyses to further identify secondary association signals within the identified loci, nor did we perform functional studies for the identified variants. Further research is therefore needed to unravel the exact biological mechanism behind the observed associations. The fact that various TPOAb assays were used across the participating cohorts could lead to bias. We therefore used TPOAb-positivity cut-off values as provided by the respective assay manufacturer, instead of using one fixed cut-off value. This is also of clinical importance as in clinical practice most institutions rely on the TPOAb-positivity cut-off as provided by the assay manufacturer. Furthermore, we did not detect heterogeneity in our results, supporting the fact that results obtained with different assays can be combined across cohorts using the z-score based meta-analysis. Finally, as AITD coincides with other autoimmune diseases, our results could be driven by indirect associations with other autoimmune diseases. However, AITD is the most common autoimmune disease in the general population. We furthermore show that carriage of multiple risk alleles is associated with an increased risk of thyroid dysfunction, which underlines the clinical importance of our findings.

In conclusion, this first GWAS for TPOAbs identified five newly associated loci, three of which were also associated with clinical thyroid disease. Furthermore, we show that carriage of multiple risk variants is not only associated with a substantial increased risk of TPOAb-positivity, but also with a higher risk of increased TSH levels (including subclinical and overt hypothyroidism) and a lower risk of goiter. These genetic markers not only help to identify large groups in the general population with an increased risk of TPOAb-positivity, but may also predict which TPOAb-positive persons are particularly at risk of developing clinical thyroid disease.

## Materials and Methods

### Study cohorts

For the TPOAb GWAS stage 1 and 2 analyses, and the hypothyroidism, hyperthyroidism and goiter analyses, individuals were recruited from 16 independent community-based and family studies. For the Graves' disease analyses, cases were recruited from the United Kingdom Graves' disease cohort and controls from the British 1958 Birth Cohort. Thyroid cancer cases and controls were recruited from the Nijmegen and Ohio thyroid cancer cohorts. A detailed description of the original cohorts contributing samples is provided in Table 1 and in the Supplementary Material. All participants provided written informed consent and protocols were approved by the institutional review boards or research ethics committees at the respective institutions, and conducted according to the Declaration of Helsinki.

### Phenotype definitions

Serum TPOAb levels were determined with a range of assays. TPOAb-positives were defined as subjects with TPOAb levels above the assay-specific TPOAb-positivity cut-off, as defined by the manufacturer (Table 1). Serum TSH and free thyroxine (FT4) levels were determined using a range of assays (Table 1). Assay-specific TSH and FT4 reference ranges were used, as provided by the manufacturer (Table 1). Overt hypothyroidism was defined as a high TSH (i.e., a TSH level above the TSH reference range) and a low FT4. Increased TSH was defined as a high TSH, including persons with overt hypothyroidism or subclinical hypothyroidism (i.e., high TSH with a normal FT4). Overt hyperthyroidism was defined as a low TSH and a high FT4. Decreased TSH was defined as a low TSH, including persons with subclinical or overt hyperthyroidism.

The diagnosis of goiter is described in the Supplementary Material, and the diagnosis of Graves' disease and thyroid cancer in the respective cohorts have been described previously [41].

### Genotyping

Samples were genotyped with a range of GWAS genotyping arrays (Supplementary Table S1). Sample and SNP quality control procedures were undertaken within each study. For each GWAS, over 2.5 million SNPs were imputed using CEU samples from Phase 2 of the International HapMap project (www.hapmap.org). Genotyping procedures in the stage 2, Graves' disease and thyroid cancer populations are described in the Supplementary Material.

### Association analyses

The heritabilities of TPOAb-positivity and serum TPOAb levels were estimated, as described in the Supplementary Material.

In stage 1, we performed a GWAS on TPOAb-positivity as well as a GWAS on continuous TPOAb levels. Persons taking thyroid medication were excluded. Each SNP was tested for association with TPOAb-positivity using logistic regression analyses, adjusting for age and sex. For cohorts with family structure, we approximated the probability of being affected with a linear mixed model adjusting for age and sex. The produced model was used to predict the expected proportion of “risk” (effective) alleles in cases and controls, hence giving the means to estimate odds ratios. Only unrelated individuals were considered for the SardiNIA cohort. For the GWAS of continuous TPOAb levels, samples with a TPOAb level lower than the minimum TPOAb assay detection limit (Table 1) were excluded. TPOAb levels were natural log-transformed, and sex-specific, age adjusted standardized residuals were calculated. Each SNP was tested for association with these TPOAb level residuals using linear regression analyses (additive model), correcting for relatedness in studies with family structure. See Supplementary Table S1 for the software used for these analyses.

Before meta-analysis, SNPs with a minor allele frequency (MAF) <1% or a low imputation quality were excluded (Supplementary Material), after which the results of each GWAS were combined in a population size weighted z-score based meta-analysis using METAL [71]. Genomic control was applied to individual studies if λ>1.0.

In stage 2, we followed-up stage 1 GWAS significant SNPs, as well as promising SNPs not reaching GWAS significance, in an attempt to reach GWAS significant associations by increasing sample size (Supplementary Material). Results from stage 1 and 2 were combined in a population size weighted z-score based meta-analysis using METAL [71]. A z-score based meta-analysis was used to reduce bias that might be induced by different assays. As this method does not provide betas, and we wanted to provide a rough estimate of the actual effect sizes for convenience, we calculated betas using the fixed effects (inverse variance based) meta-analysis method. Heterogeneity was tested, applying bonferroni based *P*-value thresholds of *P* = 0.004 for the TPOAb-positivity analyses and *P* = 0.005 for the TPOAb level analyses.

All studies assessed and, if present, corrected for population stratification using principal-component analysis (PCA) and/or multidimensional-scaling (MDS), with the exception of SardiNIA and ValBorbera where the high isolation substantiates a lack of stratification (Table S1) [72], [73]. Lambda values were all ∼1, indicating that population stratification was overall properly accounted for (Table S1). To fully remove residual effects, we applied genomic correction to studies were lambda was >1. The final meta-analyses reported a lambda of 1.01 for both the TPOAb-positivity and the TPOAb level GWAS, thus no genomic correction was applied.

The variances explained by the GWAS significant SNPs were calculated. We subsequently studied the individual as well as the combined effects of the GWAS significant SNPs on the risk of clinical thyroid disease, as specified in the Supplementary Material. In short, to study combined effects, a genetic risk score was calculated for every person as the weighted sum of TPOAb risk alleles. The associations between the individual SNPs, genetic risk scores and the risk of abnormal thyroid function tests were studied using logistic regression analyses. Logistic regression analyses were used to study the associations with goiter, Graves' disease and thyroid cancer (Supplementary Material). The results of each study were combined in a population size weighted z-score based meta-analysis using METAL [71].

Various bioinformatic tools were searched for evidence for functional relevance of the GWAS significant SNPs and pathway analyses were performed on the Stage 1 lead SNPs (see Supplementary Material).

## Supporting Information

Figure S1 TPOAb level distributions in persons with detectable TPOAb levels in stage 1 and 2 populations.
(PPTX)

Figure S2 Quantile-quantile (QQ) plots for the TPOAb-positivity and TPOAb level stage 1 meta-analyses.
(PPTX)

Figure S3 Manhattan plots for stage 1 meta-analyses for TPOAb-positivity (a) and TPOAb levels (b). SNPs are plotted on the x-axis according to their chromosomal position against TPOAb-positivity (a) or TPOAb levels (b) (shown as – log_10_ *P* value) on the y-axis. The horizontal grey line indicates the threshold for genome-wide statistical significance (*P*<5×10^−8^). Genome-wide significant associations were observed near *TPO* (Chr 2p25; *P* = 1.5×10^−12^), at *ATXN2* (Chr 12q24.1; *P* = 1.6×10^−9^) and near *HCP5* (Chr 6p21.3; *P* = 4.1×10^−8^) for TPOAb-positivity, and near *TPO* (Chr 2p25; *P* = 5.4×10^−13^) and at *ATXN2* (Chr 12q24.1; *P* = 1.1×10^−8^) for TPOAb levels.
(PPTX)

Figure S4 Regional association plots of stage 1 lead loci for TPOAb-positivity (panels a-m). The y-axis on the left indicates the – log_10_ *P* value for the association with TPOAb –positivity. SNPs are plotted on the x-axis according to their chromosomal position. The most significant stage 1 SNP is indicated in purple. The combined stage 1 and 2 result of this SNP is indicated in yellow. The SNPs surrounding the most significant SNP are color-coded to reflect their LD with this SNP. Symbols reflect functional genomic annotation, as indicated in the legend. The blue y-axes on the right of each plot indicate the estimated recombination rates (based on HapMap Phase II); the bottom of each panel shows the respective annotated genes at the locus and their transcriptional direction. Mb, megabases.
(PPTX)

Figure S5 Regional association plots of stage 1 lead loci for TPOAb levels (panels a-j). The y-axis on the left indicates the – log_10_ *P* value for the association with TPOAb levels. SNPs are plotted on the x-axis according to their chromosomal position. The most significant stage 1 SNP is indicated in purple. The combined stage 1 and 2 result of this SNP is indicated in yellow. The SNPs surrounding the most significant SNP are color-coded to reflect their LD with this SNP. Symbols reflect functional genomic annotation, as indicated in the legend. The blue y-axes on the right of each plot indicate the estimated recombination rates (based on HapMap Phase II); the bottom of each panel shows the respective annotated genes at the locus and their transcriptional direction. Mb, megabases.
(PPTX)

Figure S6 GRAIL results for the stage 1 TPOAb-positivity and TPOAb level lead SNPs. GRAIL circle plot of locus connectivity where each locus is plotted in a circle, where significant connections (*P*<0.05) based on PubMed abstracts are drawn spanning the circle. Analyses were based on the 20 stage 1 TPOAb-positivity and TPOAb level lead SNPs.
(PPTX)

Table S1 Study sample genotyping, quality control and association analyses for stage 1 populations.
(DOCX)

Table S2 Associations of stage 1 lead SNPs with TPOAb-positivity in stage 1 and 2.
(DOCX)

Table S3 Associations of stage 1 lead SNPs with serum TPOAb levels in stage 1 and 2.
(DOCX)

Table S4 Stage 1 TPOAb-positivity and TPOAb level meta-analyses results for GWAS significant SNPs reported in previous GWAS on thyroid related phenotypes.
(XLSX)

Table S5 Genetic risk score and the risk of increased TSH levels.
(DOCX)

Table S6 Newly identified TPOAb associated loci and the risk of thyroid cancer.
(DOCX)

Table S7 Top IPA associated networks for the Stage 1 TPOAb-positivity and TPOAb level lead SNPs.
(DOCX)

Table S8 Top IPA associated canonical pathways for the Stage 1 TPOAb-positivity and TPOAb level lead SNPs.
(DOCX)

Text S1 Supplementary methods.
(DOCX)
