# Supplementary material for: Identification of Novel Genetic Loci Associated with Thyroid Peroxidase Antibodies and Clinical Thyroid Disease
Source: PLoS Genet. 2014 Feb 27;10(2):e1004123. doi: 10.1371/journal.pgen.1004123 (PMC3937134; doi:10.1371/journal.pgen.1004123)
Supplement: Table S1 — Study sample genotyping, quality control and association analyses for stage 1 populations. (DOCX) [file pgen.1004123.s007.docx]

|  | Genotyping | | Sample QC | | SNP QC | | | | Imputation | | |  |  | Case-control analyses | | Continuous analyses | |
| --- | --- | --- | --- | --- | --- | --- | --- | --- | --- | --- | --- | --- | --- | --- | --- | --- | --- |
| Study | N | Platform | Call rate | Exclusion criteria | Call rate | HWE | MAF | N of SNPs for imputation | Reference panel | Software | N of SNPs in analyses | Covariates used in all analyses | Population stratification  correction or assessment^a^ | Software | λ | Software | λ |
| BHS | 1366 | Illumina Human 610 quad | ≥ 0.95 | Relatedness and duplicates | ≥ 0.90 | *P* >10^-6^ | ≥ 0.01 | 549,294 | HapMap II, CEU, Build 36 | MACH | 2,543,887 | Age, sex, asthma status | Yes  (no stratification) | GenABEL | 1.01 | GenABEL | 1.01 |
| CHS | 3271 | Illumina 370CNV BeadChip | ≥ 0.95 | Non-European descent | ≥ 0.97 | *P* >10^-5^ | - | 306,655 | HapMap II, CEU, Build 36 | BIMBAM v0.99 | 2,573,510 | Age, sex, clinic site | Yes (Exclusion non-European descent) | R | 1.03 | R | 1.01 |
| HBCS | 1728 | Illumina 610k | ≥ 0.95 | Relatedness and duplicates | ≥ 0.95 | *P* >10^-5^ | ≥ 0.01 | 509,948 | HapMap II, CEU, Build 36 | MACH | 2,543,887 | Age, sex | Yes  (no stratification) | Plink and ProbABEL | 1.00 | Plink and ProbABEL | 1.00 |
| KORA  **Table S1. Study sample genotyping, quality control and association analyses for stage 1 populations.** | 1814 | Affymetrix 6.0 (1000K) | ≥ 0.95 | Relatedness and duplicates | ≥ 0.93 | *P* >10^-6^ | ≥ 0.01 | 909,622 | HapMap II, CEU, Build 36 | IMPUTE | 2,743,205 | Age, sex | Yes (PCA) | SNPTEST | 1.03 | SNPTEST | 1.01 |
| NBS | 1980 | Illumina  370 CNV  BeadChip | ≥ 0.95 | Non-Caucasian ancestry | ≥ 0.95 | *P* >10^-6^ | ≥ 0.01 | 311,918 | HapMap II, CEU, Build 36 | IMPUTE | 2,542,995 | Age, sex | Yes (Exclusion non-Caucasian ancestry) | SNPTEST | 1.01 | SNPTEST | 1.00 |
| RS | 5974 | Illumina HumanHap 550K v.3 | ≥ 0.98 | Relatedness and duplicates | ≥ 0.98 | *P* >10^-6^ | ≥ 0.01 | 512,349 | HapMap II, CEU, Build 36 | MACH | 2,543,887 | Age, sex | Yes  (no stratification) | Mach2dat   (GRIMP)* | 1.02 | Mach2qtl (GRIMP)* | 1.01 |
| SardiNIA | 4694 | Affymetrix 10K, 500K, 6.0 | >0.95 | NA | >0.90 (10/500K) >0.95(6.0K) | *P* >10^-6^ | >0.05 (10K,500K) >0.01(6.0) | 731,209 | HapMap II, CEU, Build 36 | MACH | 2,353,985 | Age, sex | No (high isolation) | Mach2dat | 1.08 | MERLIN  (--fastassoc) | 1.13 |
| SHIP | 4081 | Affymetrix SNP Array 6.0 | ≥ 0.92 | Duplicates | - | - | - | 869,224 | HapMap II, CEU, Build 36 | IMPUTE | 2,748,910 | Age, sex | Yes  (no stratification) | SNPTEST | 1.05 | SNPTEST | 1.01 |
| SHIP-Trend | 986 | Illumina Human Omni 2.5 | ≥ 0.94 | Duplicates | ≥ 0.90 | *P* >10^-4^ | - | 1,782,967 | HapMap II, CEU, Build 36 | IMPUTE | 3,437,411 | Age, sex | Yes  (no stratification) | SNPTEST | 1.06 | SNPTEST | 0.88 |
| TwinsUK | 2455 | Illumina Hap300, Hap550, Hap610 | ≥ 0.95 | Relatedness and duplicates | ≥ 0.90 | *P* >10^-6^ | ≥ 0.01 | 295,702 | HapMap II, CEU, Build 36 | IMPUTE | 2,544,232 | Age | Yes (Exclusion non-European descent) | GenABEL | 1.00 | GenABEL | 1.00 |
| ValBorbera | 1664 | Illumina 370K - HumanCN370-Quadv3 | > 0.95 | Relatedness and duplicates | ≥ 0.90 | P >10^-4^ | ≥ 0.01 | 324,326 | HapMap II, CEU, Build 36 | MACH | 2,412,004 | Age, sex | No (high isolation) | GenABEL | 1.01 | GenABEL | 1.01 |

^a^ Using PCA (Principal-components analysis) or MDS (Multidimensional-scaling)
