# Supplementary material for: Identification of Novel Genetic Loci Associated with Thyroid Peroxidase Antibodies and Clinical Thyroid Disease
Source: PLoS Genet. 2014 Feb 27;10(2):e1004123. doi: 10.1371/journal.pgen.1004123 (PMC3937134; doi:10.1371/journal.pgen.1004123)
Supplement: Table S2 — Associations of stage 1 lead SNPs with TPOAb-positivity in stage 1 and 2. (DOCX) [file pgen.1004123.s008.docx]

| **Table S2. Associations of stage 1 lead SNPs with TPOAb-positivity in stage 1 and 2** | | | | | | | | | | | |
| --- | --- | --- | --- | --- | --- | --- | --- | --- | --- | --- | --- |
|  |  |  | Alleles | | *Stage 1* Up to 1769 cases +  16,528 controls | | *Stage 2* Up to 922 cases +  8068 controls | | *Stage 1 + 2*  Up to 2691 cases +  24,596 controls | |  |
| SNP | Chr | Position (Build 36) | Risk | Other | OR (95% CI) | *P* | OR  (95% CI) | *P* | OR (95% CI) | *P* | Het *P* |
| rs11675434 | 2 | 1386822 | T | C | 1.20  (1.13-1.27) | 1.5x10^-12^ | 1.28 (1.14-1.42) | 1.9x10^-5^ | 1.21 (1.15-1.28) | 1.5x10^-16^ | 0.08 |
| rs653178 | 12 | 110492139 | C | T | 1.14  (1.08-1.20) | 1.6x10^-9^ | 1.13 (1.02-1.25) | 0.04 | 1.14 (1.08-1.19) | 9.9x10^-10^ | 0.29 |
| rs3094228 | 6 | 31537906 | C | T | 1.33  (1.20-1.47) | 4.1x10^-8^ | 1.08 (0.94-1.23) | 0.31 | 1.23  (1.13-1.33) | 3.8x10^-7^ | 0.08 |
| rs301799 | 1 | 8411889 | C | T | 1.13  (1.07-1.20) | 1.2x10^-7^ | 1.02 (0.90-1.16) | 0.72 | 1.11 (1.06-1.17) | 2.5x10^-6^ | 0.39 |
| rs10944479 | 6 | 90937114 | A | G | 1.31  (1.16-1.47) | 1.4x10^-7^ | 1.16 (1.01-1.34) | 0.04 | 1.25 (1.14-1.37) | 4.0x10^-8^ | 0.35 |
| rs1894407 | 6 | 32895014 | C | A | 1.19  (1.12-1.27) | 1.5x10^-7^ | 1.10 (0.96-1.25) | 0.13 | 1.17 (1.11-1.24) | 1.2x10^-7^ | 0.41 |
| rs4811340 | 20 | 50443164 | G | C | 1.14  (1.08-1.21) | 2.1x10^-7^ | 1.08 (0.95-1.24) | 0.28 | 1.13 (1.07-1.20) | 5.6x10^-7^ | 0.09 |
| rs11081453 | 18 | 8990246 | T | C | 1.26  (1.14-1.40) | 1.5x10^-6^ | 1.01 (0.89-1.15) | 0.98 | 1.16 (1.07-1.26) | 7.7x10^-5^ | 0.10 |
| rs11602677 | 11 | 122681726 | A | G | 1.19  (1.11-1.27) | 1.7x10^-6^ | 0.93 (0.81-1.07) | 0.34 | 1.13 (1.07-1.20) | 3.3x10^-4^ | 0.08 |
| rs4889009 | 16 | 78257948 | G | C | 1.14  (1.08-1.22) | 1.8x10^-6^ | 1.14 (1.00-1.29) | 0.05 | 1.14 (1.08-1.21) | 3.3x10^-7^ | 0.82 |
| rs353648 | 11 | 35146865 | T | G | 1.28  (1.15-1.41) | 3.2x10^-6^ | 1.03 (0.86-1.24) | 0.83 | 1.19 (1.09-1.30) | 1.2x10^-4^ | 0.24 |
| rs9359543 | 6 | 83592789 | T | C | 1.32  (1.17-1.50) | 3.7x10^-6^ | 1.04 (0.88-1.22) | 0.71 | 1.21 (1.10-1.34) | 6.2x10^-5^ | 0.05 |
| rs879564 | 19 | 4719917 | A | G | 1.14  (1.07-1.21) | 3.9x10^-6^ | 1.20 (1.05-1.38) | 0.01 | 1.15 (1.09-1.22) | 1.1x10^-7^ | 0.89 |

Chr., chromosome
Het *P*, heterogeneity *P-*value (significance threshold *P* = 0.004).
All analyses adjusted for age and gender
