# Supplementary material for: Identification of Novel Genetic Loci Associated with Thyroid Peroxidase Antibodies and Clinical Thyroid Disease
Source: PLoS Genet. 2014 Feb 27;10(2):e1004123. doi: 10.1371/journal.pgen.1004123 (PMC3937134; doi:10.1371/journal.pgen.1004123)
Supplement: Table S3 — Associations of stage 1 lead SNPs with serum TPOAb levels in stage 1 and 2. (DOCX) [file pgen.1004123.s009.docx]

| **Table S3. Associations of stage 1 lead SNPs with serum TPOAb levels in stage 1 and 2** | | | | | | | | | | | |
| --- | --- | --- | --- | --- | --- | --- | --- | --- | --- | --- | --- |
|  |  |  | Alleles | | *Stage 1* Up to 12,353 subjects | | *Stage 2* Up to 8159 subjects | | *Stage 1 + 2* Up to 20,512 subjects | |  |
| SNP | Chr | Position (Build 36) | Risk | Other | *β* (SE) | *P* | *β* (SE) | *P* | *β* (SE) | *P* | Het *P* |
| rs11675434 | 2 | 1386822 | T | C | 0.0182 (0.0048) | 5.4x10^-13^ | 0.0429 (0.0229) | 0.01 | 0.0202 (0.0046) | 7.4x10^-13^ | 0.06 |
| rs653178 | 12 | 110492139 | C | T | 0.0144 (0.0047) | 1.1x10^-8^ | 0.0607 (0.0224) | 0.18 | 0.0147 (0.0045) | 1.3x10^-7^ | 0.14 |
| rs3094228 | 6 | 31537906 | C | T | 0.0179 (0.0062) | 1.6x10^-7^ | 0.1730 (0.0342) | 0.84 | 0.0240 (0.0059) | 5.8x10^-5^ | 0.31 |
| rs1230666 | 1 | 113974933 | A | G | 0.0241 (0.0067) | 7.9x10^-8^ | 0.0495 (0.0321) | 0.02 | 0.0269 (0.0064) | 1.8x10^-8^ | 0.23 |
| rs9277555 | 6 | 33163583 | G | A | 0.0198 (0.0052) | 2.2x10^-7^ | 0.0322 (0.0299) | 0.20 | 0.0199 (0.0050) | 5.8x10^-7^ | 0.36 |
| rs1273522 | 19 | 15501972 | G | A | 0.0211 (0.0053) | 2.7x10^-7^ | -0.0047 (0.0231) | 0.76 | 0.0195 (0.0050) | 2.0x10^-5^ | 0.01 |
| rs2010099 | 3 | 125782947 | C | T | 0.0197 (0.0080) | 7.5x10^-7^ | 0.0565 (0.0360) | 0.01 | 0.0240 (0.0076) | 3.1x10^-8^ | 0.23 |
| rs8008408 | 14 | 34913567 | A | G | 0.0285 (0.0081) | 2.1x10^-6^ | 0.0301 (0.0353) | 0.81 | 0.0261 (0.0077) | 1.2x10^-4^ | 0.19 |
| rs17048919 | 3 | 8287555 | G | C | 0.0401 (0.0077) | 2.4x10^-6^ | 0.0004 (0.0313) | 0.27 | 0.0344 (0.0073) | 3.9x10^-3^ | 0.03 |
| rs1192621 | 1 | 18209837 | A | T | 0.0229 (0.0052) | 3.4x10^-6^ | 0.0026 (0.0247) | 0.73 | 0.0279 (0.0050) | 8.2x10^-5^ | 0.11 |

Chr., chromosome
Het *P*, heterogeneity *P-*value (significance threshold *P* = 0.005).
Effects are expressed in SD of natural logarithm transformed serum TPOAb level, adjusted for age and gender.
