# Supplementary material for: Identification of Novel Genetic Loci Associated with Thyroid Peroxidase Antibodies and Clinical Thyroid Disease
Source: PLoS Genet. 2014 Feb 27;10(2):e1004123. doi: 10.1371/journal.pgen.1004123 (PMC3937134; doi:10.1371/journal.pgen.1004123)
Supplement: Table S5 — Genetic risk score and the risk of increased TSH levels. (DOCX) [file pgen.1004123.s011.docx]

| **Table S5. Genetic risk score and the risk of increased TSH levels** | | | |
| --- | --- | --- | --- |
| GRS Quartile | % Increased TSH levels (N cases/total) | OR (95% CI)^a^ | *P* value |
| 1 (reference) | 4.2 % (212 / 5051) | - | - |
| 2 | 5.7 % (290 / 5050) | 1.37 (1.13-1.66) | 1.5 x 10^-3^ |
| 3 | 5.8 % (299 / 5185) | 1.45 (1.20-1.75) | 6.8 x 10^-4^ |
| 4 | 6.2 % (309 / 5013) | 1.51 (1.26-1.82) | 2.9 x 10^-6^ |

GRS, genetic risk score (based on rs11675434, rs653178, rs10944479, rs1230666, rs2010099).
^a^ Adjusted for age and gender
