# Supplementary material for: Identification of Novel Genetic Loci Associated with Thyroid Peroxidase Antibodies and Clinical Thyroid Disease
Source: PLoS Genet. 2014 Feb 27;10(2):e1004123. doi: 10.1371/journal.pgen.1004123 (PMC3937134; doi:10.1371/journal.pgen.1004123)
Supplement: Table S6 — Newly identified TPOAb associated loci and the risk of thyroid cancer. (DOCX) [file pgen.1004123.s012.docx]

| **Table S6. Newly identified TPOAb associated loci and the risk of thyroid cancer** | | | | | | | | | |
| --- | --- | --- | --- | --- | --- | --- | --- | --- | --- |
|  |  | Alleles | | Nijmegen cohort (154 cases/ 2019 controls) | | Ohio cohort (179 cases / 190 controls) | | Combined (333 cases / 2209 controls) | |
| Nearby Gene | SNP | Risk | Other | OR (95% CI) | *P* | OR (95% CI) | *P* | OR (95% CI) | *P* |
| *TPO* | rs11675434 | T | C | 1.01  (0.68-1.50) | 0.97 | 1.04 (0.75-1.43) | 0.83 | 1.03 (0.80-1.32) | 0.85 |
| *ATXN2* | rs653178 | C | T | 1.36  (0.87-2.12) | 0.17 | 1.30 (0.95-1.77) | 0.10 | 1.32 (1.02-1.70) | 0.03 |
| *BACH2* | rs10944479 | A | G | 1.69  (1.14-2.53) | 0.01 | 0.86 (0.56-1.33) | 0.50 | 1.24 (0.92-1.66) | 0.15 |
| *MAGI3* | rs1230666 | A | G | 0.99  (0.63-1.55) | 0.95 | 0.92 (0.57-1.52) | 0.75 | 0.89 (0.64-1.25) | 0.51 |
| *KALRN* | rs2010099 | C | T | 1.22  (0.77-1.94) | 0.40 | 1.51 (0.97-2.34) | 0.07 | 1.37 (0.99-1.88) | 0.06 |

Adjusted for age and gender
