# Supplementary material for: Identification of Novel Genetic Loci Associated with Thyroid Peroxidase Antibodies and Clinical Thyroid Disease
Source: PLoS Genet. 2014 Feb 27;10(2):e1004123. doi: 10.1371/journal.pgen.1004123 (PMC3937134; doi:10.1371/journal.pgen.1004123)
Supplement: Table S7 — Top IPA associated networks for the Stage 1 TPOAb-positivity and TPOAb level lead SNPs. (DOCX) [file pgen.1004123.s013.docx]

| **Table S7. Top IPA associated networks for the stage 1 TPOAb-positivity and TPOAb level lead SNPs** | | | |
| --- | --- | --- | --- |
| **Associated Network Functions** | **Score** | **Focus  Molecules** | **Molecules in Network** |
| Cell Death and Survival, Renal Necrosis/Cell Death, Cellular Movement | 24 | 10 | ARHGEF1, **BACH2**, CD3, **CD44**, CYB5R3, DOK3, DR4/5, EZH2, FUCA1, GZMK, **HCP5**, Igm, ITGB1, Jnk, **KALRN**, **MAF**, **MAGI3**, NFkB (complex), **NFKBIA**, PELI3, PRMT2, RAC1, **RERE**, RIOK3, RTKN, SCFD1, SUMO4,Taok2,TNIP3, **TPO**, VOPP1, ZFAND6, **ZFP64**, Zfp125, ZMYND11 |
| Carbohydrate Metabolism, Molecular Transport, Small Molecule Biochemistry | 5 | 3 | **ATXN2**, CD36, FABP4, FBP1, **FEM1A**, FFAR4, GCG, HADHA, IGF2, Ins1, INSR, IRS2, LCP1, LRP5, MIF, **NDUFV2**, NKX2-2, NOS3, PDE3B, PDK4, PDX1, PIK3R2, PLA2G1B, PPARGC1A, PRKCI, PTPN1, PTPRN, RPS6KB1, SIRT1, SIRT6, SLC2A1, SLC2A4, TFAM, UCP2, UCP3 |
| Cellular Growth and Proliferation, Respiratory System Development and Function, Immunological Disease | 3 | 1 | **HLA-DPB1**, MAGEA3/MAGEA6 |
| Immunological Disease, Infectious Disease, Cell Morphology | 2 | 1 | CD63, CD82, EBI3, **HLA-DOB**, Hla-Drb, IL27, mir-223 |

Physical function analysis for the 20 stage 1 lead SNPs using IPA (Ingenuity Pathway Analysis). Four networks were generated which are ordered by a score denoting significance.
