# Supplementary material for: Identification of Novel Genetic Loci Associated with Thyroid Peroxidase Antibodies and Clinical Thyroid Disease
Source: PLoS Genet. 2014 Feb 27;10(2):e1004123. doi: 10.1371/journal.pgen.1004123 (PMC3937134; doi:10.1371/journal.pgen.1004123)
Supplement: Table S8 — Top IPA associated canonical pathways for the Stage 1 TPOAb-positivity and TPOAb level lead SNPs. (DOCX) [file pgen.1004123.s014.docx]

| **Table S8. Top IPA associated canonical pathways for the stage 1 TPOAb-positivity and TPOAb level lead SNPs** | |
| --- | --- |
| **Canonical Pathways** | ***P*-value** |
| OX40 Signaling Pathway | 7.6 x 10^-5^ |
| Antigen Presentation Pathway | 5.9 x 10^-4^ |
| Autoimmune Thyroid Disease Signaling | 1.0 x 10^-3^ |
| Cytotoxic T Lymphocyte-mediated Apoptosis of Target Cells | 2.8 x 10^-3^ |
| Allograft Rejection Signaling | 2.9 x 10^-3^ |

Top Canonical Pathways for the 20 stage 1 lead SNPs using IPA (Ingenuity Pathway Analysis).
