# Supplementary material for: Identification of Novel Genetic Loci Associated with Thyroid Peroxidase Antibodies and Clinical Thyroid Disease
Source: PLoS Genet. 2014 Feb 27;10(2):e1004123. doi: 10.1371/journal.pgen.1004123 (PMC3937134; doi:10.1371/journal.pgen.1004123)
Supplement: Text S1 — Supplementary methods. (DOCX) [file pgen.1004123.s015.docx]

**Supplementary Text S1**

**Cohort descriptions

Stage 1 cohorts

Busselton Health Study (BHS):** The BHS includes a series of cross-sectional health surveys carried out since 1966 of residents of Busselton, a rural town with a predominantly Caucasian population, located in the southwest of Western Australia [74]. In 1994-5, there was a follow-up study of people who had participated in previous studies. Participants completed a health questionnaire, underwent physical examination, and gave a venous blood sample in the morning after an overnight fast.

**Cardiovascular Health Study (CHS):** The CHS is a population-based cohort study of risk factors for coronary heart disease and stroke in adults aged 65 years or older conducted across four field centers in the United States [75]. The original cohort of 5,201 persons consisting of 4,925 Caucasians was recruited in 1989-1990 from a random sample of people on Medicare eligibility lists. CHS participants completed standardized clinical examinations and questionnaires at study baseline and at nine annual follow-up visits.

**Helsinki Birth Cohort Study (HBCS):** The HBCS is composed of 8,760 individuals born between the years 1934-44 in one of the two main maternity hospitals in Helsinki, Finland. Between 2001 and 2003, a randomly selected sample of 928 males and 1,075 females participated in a clinical follow-up study with a focus on cardiovascular, metabolic and reproductive health, cognitive function and depressive symptoms. Detailed information on the selection of the HBCS participants and on the study design can be found elsewhere [76,77]. Details of the thyroid studies have been described for women and are similar for men in the cohort [78].

**KORA:** The KORA discovery study is a population-based cohort study including 1287 probands aged 32 to 79 from the Cooperative Health Research in the Region of Augsburg Study (KORA F4, Southern Germany), which has been described in detail previously [79]. All individuals were of European ancestry.

**Nijmegen Biomedical Study (NBS):** The NBS is a population-based survey on lifestyle and medical history in 9350 men and women living in Nijmegen, The Netherlands. Rationale and design have been described previously [80].

**Rotterdam Study (RS):** The Rotterdam Study is a prospective population-based cohort study on determinants of chronic diseases in the elderly, which has been described previously [81,82]. The study comprised 7983 men and women aged 55 years and over, living in a district of Rotterdam, The Netherlands.

**SardiNIA:** The SardiNIA study consists of 6,148 volunteers, males and females, ages 14–102 years, recruited and phenotyped from a cluster of four towns in the Ogliastra province of Sardinia [73]. The local ethical committee approved the study protocol and all participants provided a written informed consent. Genotyping was performed in 4,694 individuals using either the Affymetrix 10K, Affymetrix 500K or Affymetrix 6.0 arrays, and missing genotypes imputed using a within-families approach, as previously described [83].

**Study of Health in Pomerania (SHIP):** The SHIP is a cross-sectional survey in West Pomerania, the north-east area of Germany [84,85]. A sample from the population aged 20 to 79 years was drawn from population registries. First, the three cities of the region (with 17,076 to 65,977 inhabitants) and the 12 towns (with 1,516 to 3,044 inhabitants) were selected, and then 17 out of 97 smaller towns (with less than 1,500 inhabitants), were drawn at random. Second, from each of the selected communities, subjects were drawn at random, proportional to the population size of each community and stratified by age and gender. Only individuals with German citizenship and main residency in the study area were included. Finally, 7,008 subjects were sampled, with 292 persons of each gender in each of the twelve five-year age strata. In order to minimize drop-outs by migration or death, subjects were selected in two waves. The net sample (without migrated or deceased persons) comprised 6,267 eligible subjects. The SHIP population finally comprised 4,308 participants (corresponding to a final response of 68.7%).

**SHIP–Trend:** The SHIP-Trend is a longitudinal population-based cohort study assessing the prevalence and incidence of common, population relevant diseases and their risk factors [85]. Baseline examinations have started in 2008 and were finished in 2012. The study region is essentially the same as the study region of the initial SHIP cohort. The sample was drawn randomly from population registries.

**TwinsUK:** The TwinsUK cohort consists of 2455 female twins of Western European/UK ancestry, aged 18–82 years, from St Thomas’ UK Adult Twin Registry, a volunteer sample recruited in the United Kingdom without selection for particular traits, which has previously been shown to be representative of singleton populations and the UK population in general [86].

**Val Borbera:** The INGI‐Val Borbera population is a collection of 1,664 genotyped samples collected in the Val Borbera Valley, a geographically isolated valley located within the Appennine Mountains in Northwest Italy [87]. The valley is inhabited by about 3,000 descendants from the original population, living in 7 villages along the valley and in the mountains. Participants were healthy people 18-102 years of age that had at least one grandfather living in the valley.

**Stage 2 cohorts**

**Asklepios:** The Asklepios Study is a longitudinal population study focusing on better understanding of –and the interplay between- cardiovascular function and human aging with an eventual goal of developing better risk prevention models [88]. The 2524 participants are a population-representative cohort of 35-55 year old men and women, free from cardiovascular disease at study initiation (2002), randomly sampled from the twinned Belgian communities of Erpe-Mere and Nieuwerkerken.

**CARLA:** The CARLA study is an ongoing cohort study of a representative sample of the inhabitants of the city of Halle, eastern Germany, comprising 1,779 men and women aged 45–83 years at baseline [89]. The baseline examination took place between December 2002 and January 2006. A multi-step recruitment strategy aimed to achieve a high response rate. The ﬁnal response rate after subtracting exclusions (individuals who were deceased prior to the invitation, had moved away, or were unable to participate due to illness) was 64.1%.

**Exeter Family of Childhood Health (EFSOCH):** The EFSOCH is a consecutive birth cohort consisting of children born between 2000 and 2004 in central Exeter, UK, and their parents [90]. Both parents attended a study visit at 28 weeks of gestation, at which DNA was collected and a fasting blood sample was taken for biochemical assays. In addition, a sample of mothers attended a follow-up visit at a median of 5 years post-pregnancy, at which a further fasting blood sample was taken. 1289 fathers and post-pregnancy mothers were included in the stage 2 analyses. The effects of the 5 GWAS significant SNPs on clinical thyroid disease in pregnancy was studied in 859 pregnant mothers.

**Health2006 Study:** The Health2006 Study is a cross-sectional population-based cohort study on lifestyle factors in relation to risk of chronic disease. The participants in the Health2006 Study were drawn as a random sample from the background population aged 18 to 69 years living in 11 municipalities in the South-western part of the greater Copenhagen area. A sample of 7770 persons eligible for invitation with Danish citizenship and born in Denmark was obtained from the Danish Central Personal Register, Ministry of Internal Affairs. A total of 3471 persons entered the study and participated in the health examinations at the RCPH which took place between June 2006 and June 2008. In addition, the study was registered at www.clinical.trials.com (Unique ID: KA20060011).

**SardiNIA2:** The SardiNIA stage 2 cohort consist of 1,392 individuals from the SardiNIA cohort unrelated (kinship coefficient=0) to the individuals in Stage 1 [73,91].

**Graves’ disease and thyroid cancer cohorts**

**Graves’ disease and controls cohorts:** The United Kingdom (UK) Graves’ disease cases consisted of 2478 patients, with a mean age of 32 years and consisted of 82% females [92]. The control population consisted of 2682 geographically matched subjects drawn from the British 1958 Birth Cohort [93] (http://www.b58cgene.sgul.ac.uk/index.php). All subjects from the 1958 Birth Cohort were between the ages of 44-46 years when DNA was obtained and consisted of 50% females. All subjects were of white European ancestry with written informed consent and Ethics Committee/Institutional Review Board approval.

**Nijmegen thyroid cancer and controls cohorts:** Participants consisted of 154 individuals with non-medullary thyroid cancer (73% females, mean age at diagnosis 39.3 (SD 12.7)) and 2019 cancer-free individuals (51% females, mean age 61.5 (SD 10.3)). Affected individuals were recruited from the Department of Endocrinology, Radboud University Nijmegen Medical Centre (RUNMC), Nijmegen, The Netherlands from November 2009 to June 2010. All affected individuals were of self-reported European descent. The unaffected individuals were recruited from the Nijmegen Biomedical Study (NBS) [94]. The study was approved by the Ethical Committee and the Institutional Review Board of the RUNMC, Nijmegen, The Netherlands and all study subjects gave writ­ten informed consent.

**Ohio thyroid cancer and controls cohorts:** The study was approved by the Institutional Review Board of the Ohio State University. All subjects gave written informed consent before participation. Cases (n= 181) were histologically confirmed papillary thyroid carcinoma (PTC) patients (including traditional PTC and follicular variant PTC). These patients were admitted to the Ohio State University (OSU) Comprehensive Cancer Center. All cases were Caucasian; 39 men, 142 women, with a mean age at diagnosis of 39 (median age 38 years, range 12 to 88). Controls (n= 192) were individuals without clinically diagnosed thyroid cancer from the central Ohio area who were randomly picked from a pool of controls for genetics projects. All controls were Caucasian, 53 men, 139 women, with a mean age of 49 (median age 50 years, range 18 to 82).

**Genotyping methods**

**Stage 2 cohorts**

**Asklepios:** Genomic DNA was extracted from samples of peripheral venous blood according to standard procedures. DNA was available in all 2524 subjects. Genotypes for all 20 stage 1 SNPs were determined by Kbioscience (Unit 7, Maple Park, Hoddesdon, Herts, England UK) using the KASP on demand genotyping reagent system.

**CARLA:** Genomic DNA was extracted from samples of peripheral venous blood according to standard procedures. 1491 subjects were genotyped for rs11675434, rs653178, rs3094228, rs301799, and rs1230666. Genotypes were determined using the pre-developed TaqMan® SNP Genotyping Assays (Applied Biosystems, Darmstadt, Germany).

**EFSOCH:** Genomic DNA was extracted from samples of peripheral venous blood according to standard procedures. DNA was available in 1842 fathers and mothers with TPOAb data. Subjects were genotyped for all 20 stage 1 SNPs, except for rs3094228, rs1894407, and rs9277555. DNA samples were genotyped at KBiosciences (Hoddesdon, UK; www.kbioscience.co.uk), using their own system of fluorescence-based competitive allele-specific PCR (KASPar).
Of note, serum TSH levels were determined in 964 men and 974 pregnant women, and again in 572 women post-pregnancy. Serum FT4 levels were determined in 973 men, 974 pregnant women and 567 women post-pregnancy. Serum TSH and FT4 were analyzed using an electrochemiluminescent immunoassay, run on the Modular E170 Analyzer (Roche, Burgess Hill, UK). The manufacturer’s population reference ranges (for non-pregnant samples) were: TSH, 0.35–4.5 mIU/L; and FT4, 11–24 pmol/L. For the analyses of the pregnant women, we used reference ranges specific for the assay and 28th week of gestation based on our own set of TPOAb-negative, healthy, pregnant women (n=901): TSH, 0.49–4.21 mIU/L and FT4, 9.13–15.17 pmol/L [95].

**Health2006 Study:** Genomic DNA was extracted from samples of peripheral venous blood according to standard procedures. DNA was available in 3287 subjects with TPOAb data. Genotypes for all 20 stage 1 SNPs were determined using KBiosciences allele-specific PCR (KASPar) (KBiosciences, Hoddesdon, UK).

**SardiNIA2:** Genomic DNA was extracted from samples of peripheral venous blood according to standard procedures. DNA was available in all subjects with TPOAb data. Genotypes for rs1230666, rs3094228, rs1894407, rs9277555, rs10944479, and rs653178 were determined by ImmunoChip array, which was recently genotyped in the full Sardinia cohort but not included in the current GWAS data set. Genotyping was performed according to manufacturer's protocol, and quality control criteria have been described previously [73].

**Graves’ disease and thyroid cancer cohorts**

**Graves’ disease and controls cohorts:** Both rs10944479 and rs1230666 genotyping assays were purchased from Applied Biosystems, UK as pre-validated assays on demand. All genotyping was performed using Taqman genotyping technology on an ABI7900HT (Applied Biosystems, Warrington, UK) and all genotyping plots were independently verified by two investigators to prevent sample miscalling.

**Nijmegen thyroid cancer and controls cohorts:** Genotype data of most (N=1832) of the Dutch controls were already available at the start of the study and were obtained by Illumina HumanHap300 or HumanCNV370-Duo BeadChips [96]. Genotyping of the thyroid cancer cases (N=154) and the remaining controls (N=187) was performed by either TaqMan single nucleotide polymorphism (SNP) assays (rs11675434, rs653178, rs10944479 and rs1230666) on the 7300 ABI Real-Time polymerase chain reaction system (Applied Biosystems, CA, USA) or conventional PCR and Sanger sequencing (rs2010099).

**Ohio thyroid cancer and controls cohorts:** For both cases and controls, genomic DNA from blood samples was extracted by a standard phenol-chloroform procedure. To genotype the five SNPs, SNaPshot assay (ABI) was used as described [97]. Primer extension was carried out using the SNaPshot Multiplex Kit (ABI) according to the manufacturer's instructions. The allele analysis was performed using an ABI 3730 DNA Analyzer.

**Thyroid ultrasound measurements and diagnosis of goiter**

Thyroid ultrasound measurements in SHIP/SHIP-Trend and KORA were performed using the Ultrasound VST-Gateway 5 MHz linear array transducer (Diasonics) and the SONOLINE G50 5 MHz linear array transducer (Siemens Medical), respectively. Thyroid volume was calculated as length * width * depth * 0.479 (mL) for each lobe. Goiter was defined as a thyroid volume > 18 mL in women and > 25 mL in men, as has been described in detail previously [98].

**Heritability analyses of TPOAb-positivity and TPOAb levels**

Heritability analyses for TPOAb-positivity and TPOAb levels were performed in SardiNIA, TwinsUK and Val Borbera using SOLAR (Sequential Oligogenic Linkage Analysis Routines; <http://bioweb2.pasteur.fr/docs/solar/>). For the TPOAb levels a rank-based transformation method, using an inverse normal transformation as performed by SOLAR, was used to deal with kurtosis and skewness. For both traits, age and sex were included in the analyses. A basic model was used to estimate the additive polygenic component σ_a_^2^ and environmental component σ_e_^2^ of each trait variance due to mean effects of single alleles. Polygenic model as performed by solar quantified heritability as h^2^ = σ_a_^2^/σ_a_^2^+σ_e_^2^ and provided an estimate of the degree to which the offspring phenotypes are explained by parental phenotypes.

**Stage 1 meta-analyses**

Before meta-analysis, SNPs with a minor allele frequency (MAF) < 1% or a low imputation quality (< 0.3 for MACH and < 0.4 for IMPUTE/BIMBAM) were excluded. In addition, SNPs were excluded from the GWAS on TPOAb-positives and –negatives if (2 * N * MAF * imputation quality) < 30, where N was the total number of subjects included in the analysis of the respective SNP. The results of each GWAS were combined using a population size weighted z-score based meta-analysis, as TPOAb levels were determined with a range of different TPOAb assays (Table S1).

**Selection of stage 2 SNPs**

Stage 1 SNPs with a *P*-value ≤ 10^-5^ were separated into independent loci by clumping based on LD (r2>0.2) using PLINK. SNPs with MAF < 5 % or high heterogeneity (I^2^ ≥ 50 %) were excluded. In this way, based on I^2^ = 80.8 and heterogeneity *P*-value = 1.5x10^-6^, we excluded rs133994 from the TPOAb level analyses. Based on the MAF criteria, we excluded rs547165 (MAF = 0.01) in the TPOAb level analyses, and rs9563708 (MAF = 0.03) in the TPOAb-positivity analyses. To make the most efficient use of available resources, not all promising stage 1 SNPs had to be followed up in all stage 2 replication cohorts (see Supplementary Material S1section on genotyping methods). In this way, taking sample size and financial constraints of the stage 2 replication cohorts into account, we calculated that we were powered to establish associations at GWAS significant levels for stage 1 SNPs with *P <* 5 x 10^-6^.

**Variance explained**To evaluate the variance explained for TPOAb-positivity and serum TPOAb levels by the GWAS significant hits, we subtracted, in each GWAS study, the variance explained by the basic regression model (only including the covariates age and sex) from that explained by the full model, in which also the 5 GWAS significant SNPs were included. A weighted average of study-specific variances was calculated by combining the variance explained with weights proportional to the study sample size.

**Association analyses clinical thyroid disease**

In clinical practice, the TPOAb status (positive or negative) rather than the TPOAb level is important in decision making. As the identified 3 GWAS significant SNPs for TPOAb levels also showed associations with TPOAb-positivity (*TPO*-rs11675434*:* OR, 1.21 [95% CI, 1.15-1.28)], *P* = 1.5x10^-16^; *MAGI3*-rs1230666*:* OR, 1.23 [95% CI, 1.14-1.33], *P* = 1.5x10^-6^; *KALRN*-rs2010099*:* OR, 1.24 [95% CI, 1.12-1.37], *P* = 7.4x10^-5^), we studied the (combined) effects of all 5 SNPs on clinical thyroid disease as specified below.

**Genetic risk score and TPOAb-positivity**

A genetic risk score based on the 5 GWAS significant SNPs was calculated for every individual as the weighted sum of TPOAb-positivity risk alleles, with weights proportional to the effect estimated in the stage 1 + 2 meta-analysis. As a z-score based meta-analysis does not provide betas, we calculated betas using a fixed effects (inverse variance based) meta-analysis for TPOAb-positivity.
In each stage 2 study, we calculated genetic risk score quartiles from the global distribution of the scores. The number of TPOAb-positives and –negatives were compared between the genetic risk score quartiles, using logistic regression analyses, adjusting for age and sex. The results of each study were combined using a population size weighted z-score based meta-analysis.

**Associations with hypo- and hyperthyroidism**

The associations between genetic risk score quartiles and the risk of increased TSH levels, overt hypothyroidism, decreased TSH levels and overt hyperthyroidism were studied as well, using the same method as for the genetic risk score vs TPOAb-positivity analyses. The associations with the individual SNPs were studied as well, using logistic regression analyses, adjusting for age and sex. The results of each study were combined using a population size weighted z-score based meta-analysis. Bonferroni threshold was used to define significance of associations.

As thyroid hormone metabolism significantly changes during pregnancy [24], we additionally studied the individual and combined effects of the GWAS significant SNPs on the risk of thyroid dysfunction during pregnancy. These effects were studied in 859 pregnant women from the EFSOCH cohort, including 59 TPOAb-positives, 38 women with increased TSH and 13 women with suppressed TSH levels. As there were only 2 overt hypothyroid and 7 overt hyperthyroid women, we were unable to study the effects on overt hypo- and hyperthyroidism during pregnancy.

**Associations with goiter**

Thyroid ultrasound data were available in 3614 SHIP, 887 SHIP-Trend, and 1290 KORA subjects. Subjects using thyroid medication and pregnant women were excluded, except in SHIP-Trend in which pregnancy data were unavailable. The associations between the individual SNPs, genetic risk scores and goiter were studied using logistic regression analyses, adjusting for age, sex and body surface area (BSA). BSA was calculated as 0.007184 x (weight[kg])^0.425^ x (height[cm])^0.725^. The results of each study were combined using a population size weighted z-score based meta-analysis.

**Associations with thyroid disease in independent populations**

Since Graves’ disease is the major cause of hyperthyroidism, and considering the fact that Hashimoto’s thyroiditis and Graves’ disease co-segregate in families [17,20,21], we selected those SNPs that showed promising associations with hyperthyroidism in our meta-analyses (i.e., *P* ≤ 0.05). These SNPs were tested in an independent population of 2478 patients with Graves’ disease and 2682 controls using logistic regression analyses, adjusting for age and sex.

As both thyroid autoimmunity and abnormal TSH levels have been implicated in the development of thyroid cancer [99,100], we tested the risk of thyroid cancer for the GWAS significant SNPs in the Nijmegen and Ohio cohorts, including in total 333 cases and 2209 controls. Associations were studied using logistic regression analyses, adjusting for age and sex. The results of these studies were combined using a population size weighted z-score based meta-analysis.

**Effects of previously identified thyroid related SNPs in the stage 1 TPOAb-positivity and TPOAb level meta-analyses**

Table S4 shows the stage 1 TPOAb-positivity and TPOAb level meta-analyses results for GWAS significant SNPs reported in 15 previous GWAS on thyroid related phenotypes [36,37,41,91,101-107]. We reported a proxy (r^2^>0.8) and the relative r^2^ in case the marker was not available. The most significant associations were found for SNPs in or near *CTLA4*, *PTPN22*, *SH2B3*, and *MAF*. The rs11694732 variant, which is in LD with our top hit *TPO-*rs11675434 (r^2^=0.97 in HapMap2), was also associated with both TPOAb-positivity and TPOAb levels. However, given the higher *P-*value this association is most likely driven by *TPO-*rs11675434.

**Bioinformatics tools search for functional relevance**

For the GWAS significant SNPs, bioinformatics tools were searched for functional relevance including Gwava (www.sanger.ac.uk/sanger/StatGen_Gwava), Haploreg [108], and eQTL resources (http://eqtl.uchicago.edu). However, this did not lead to additional insights (data not shown).

**Pathway analyses**Pathway analyses were performed using the Ingenuity Pathway Analysis software tool Network (IPA; Ingenuity Systems, Ca, USA) in order to get more insight into possible pathways and networks involved. Focus genes for network inquiry were selected using the top 20 stage 1 GWAS SNPs (Tables S2 and S3). Molecules and/or relationships considered were the ones available in the IPA Knowledge Base for mammals (human, mouse or rat). Confidence filters were set to consider only relationships where the confidence is Experimentally Observed or High (Predicted). Networks were generated with a maximum size of 35 genes and allowing up to 25 networks per analysis. The networks are constructed using the IPA algorithm which generates a score as well as a *P*-value. IPA computes a score for each network according to the fit of that network to the set of focus genes. The *P*-value is calculated using the right-tailed Fisher Exact test. The 20 promising loci were mapped in the Ingenuity Knowledge base and all were considered for network construction on available interactions. In this way, Ingenuity generated 4 networks (Table S7) which included 196 nodes. There were no overlapping networks. The top 2 networks incorporated genes that code for cell death, survival and movement as well as molecular transport and carbohydrate metabolism. We assessed how genes in the networks overlap with canonical pathways in the Ingenuity database (Table S8.). The top three networks included the OX40 Signaling Pathway, Antigen Presentation Pathway and Autoimmune Thyroid Disease Signaling pathways.
The same 20 loci were used to examine functional connectivity with the Gene Relationships Among Implicated Loci Package (GRAIL; [www.broad.mit.edu/mpg/grail/](http://www.broad.mit.edu/mpg/grail/)) [109]. In short, GRAIL is a tool that searches for relationships between genes in different disease associated loci for a given set of genes or SNP’s. GRAIL mines PubMed archives looking for similarities in the published scientific text among the associated genes. Genes for text mining of the functional data source were identified using HapMap2 Release 22 CEU samples. The search included indexed abstracts from PubMed last curated on December 2006. The results of GRAIL are summarized in Figure S6. The most significant relation was found between *BACH2* and *MAF*, which were also both prominent in the cell death, survival and movement IPA pathway (Table S7). In humans, *MAF* has been shown to play a role in IL10 and IL4 expression [110] and CD4+ T-lymphocytes transcription [111].

**References:**

**74.** Walsh JP, Bremner AP, Feddema P, Leedman PJ, Brown SJ, et al. (2010) Thyrotropin and thyroid antibodies as predictors of hypothyroidism: a 13-year, longitudinal study of a community-based cohort using current immunoassay techniques. J Clin Endocrinol Metab. 95: 1095-1104.

**75.** Fried LP, Borhani NO, Enright P, Furberg CD, Gardin JM, et al. (1991) The Cardiovascular Health Study: design and rationale. Ann Epidemiol. 1: 263-276.

**76.** Barker DJ, Osmond C, Forsen TJ, Kajantie E, Eriksson JG (2005) Trajectories of growth among children who have coronary events as adults. N Engl J Med. 353: 1802-1809.

**77.** Rikkonen K, Pesonen AK, Heinonen K, Lahti J, Kajantie E, et al. (2008) Infant growth and hostility in adult life. Psychosom Med. 70: 306-313.

**78.** Kajantie E, Phillips DI, Osmond C, Barker DJ, Forsen T, et al. (2006) Spontaneous hypothyroidism in adult women is predicted by small body size at birth and during childhood. J Clin Endocrinol Metab. 91: 4953-4956.

**79.** Wichmann HE, Gieger C, Illig T (2005) KORA-gen--resource for population genetics, controls and a broad spectrum of disease phenotypes. Gesundheitswesen. 67: S26-30.

**80.** Hoogendoorn EH, Hermus AR, de Vegt F, Ross HA, Verbeek AL, et al. (2006) Thyroid function and prevalence of anti-thyroperoxidase antibodies in a population with borderline sufficient iodine intake: influences of age and sex. Clin Chem. 52: 104-111.

**81.** Hofman A, Grobbee DE, de Jong PT, van den Ouweland FA (1991) Determinants of disease and disability in the elderly: the Rotterdam Elderly Study. Eur J Epidemiol. 7: 403-422.

**82.** Hofman A, van Duijn CM, Franco OH, Ikram MA, Janssen HL, et al. (2011) The Rotterdam Study: 2012 objectives and design update. Eur J Epidemiol. 26: 657-686.

**83.** Naitza S, Porcu E, Steri M, Taub DD, Mulas A, et al. (2012) A genome-wide association scan on the levels of markers of inflammation in Sardinians reveals associations that underpin its complex regulation. PLoS Genet. 8: e1002480.

**84.** John U, Greiner B, Hensel E, Ludemann J, Piek M, et al. (2001) Study of Health In Pomerania (SHIP): a health examination survey in an east German region: objectives and design. Soz Praventivmed. 46: 186-194.

**85.** Volzke H, Alte D, Schmidt CO, Radke D, Lorbeer R, et al. (2011) Cohort profile: the study of health in Pomerania. Int J Epidemiol. 40: 294-307.

**86.** Spector TD, Williams FM (2006) The UK Adult Twin Registry (TwinsUK). Twin Res Hum Genet. 9: 899-906.

**87.** Traglia M, Sala C, Masciullo C, Cverhova V, Lori F, et al. (2009) Heritability and demographic analyses in the large isolated population of Val Borbera suggest advantages in mapping complex traits genes. PLoS One. 4: e7554.

**88.** Rietzschel ER, De Buyzere ML, Bekaert S, Segers P, De Bacquer D, et al. (2007) Rationale, design, methods and baseline characteristics of the Asklepios Study. Eur J Cardiovasc Prev Rehabil. 14: 179-191.

**89.** Greiser KH, Kluttig A, Schumann B, Kors JA, Swenne CA, et al. (2005) Cardiovascular disease, risk factors and heart rate variability in the elderly general population: design and objectives of the CARdiovascular disease, Living and Ageing in Halle (CARLA) Study. BMC Cardiovasc Disord. 5: 33.

**90.** Knight B, Shields BM, Hattersley AT (2006) The Exeter Family Study of Childhood Health (EFSOCH): study protocol and methodology. Paediatr Perinat Epidemiol. 20: 172-179.

**91.** Arnaud-Lopez L, Usala G, Ceresini G, Mitchell BD, Pilia MG, et al. (2008) Phosphodiesterase 8B gene variants are associated with serum TSH levels and thyroid function. Am J Hum Genet. 82: 1270-1280.

**92.** Manji N, Carr-Smith JD, Boelaert K, Allahabadia A, Armitage M, et al. (2006) Influences of age, gender, smoking, and family history on autoimmune thyroid disease phenotype. J Clin Endocrinol Metab. 91: 4873-4880.

**93.** Todd JA, Walker NM, Cooper JD, Smyth DJ, Downes K, et al. (2007) Robust associations of four new chromosome regions from genome-wide analyses of type 1 diabetes. Nat Genet. 39: 857-864.

**94.** Wetzels JF, Kiemeney LA, Swinkels DW, Willems HL, den Heijer M (2007) Age- and gender-specific reference values of estimated GFR in Caucasians: the Nijmegen Biomedical Study. Kidney Int. 72: 632-637.

**95.** Shields BM, Freathy RM, Knight BA, Hill A, Weedon MN, et al. (2009) Phosphodiesterase 8B gene polymorphism is associated with subclinical hypothyroidism in pregnancy. J Clin Endocrinol Metab. 94: 4608-4612.

**96.** Kiemeney LA, Sulem P, Besenbacher S, Vermeulen SH, Sigurdsson A, et al. (2010) A sequence variant at 4p16.3 confers susceptibility to urinary bladder cancer. Nat Genet. 42: 415-419.

**97.** He H, Olesnanik K, Nagy R, Liyanarachchi S, Prasad ML, et al. (2005) Allelic variation in gene expression in thyroid tissue. Thyroid. 15: 660-667.

**98.** Teumer A, Rawal R, Homuth G, Ernst F, Heier M, et al. (2011) Genome-wide association study identifies four genetic loci associated with thyroid volume and goiter risk. Am J Hum Genet. 88: 664-673.

**99.** Feldt-Rasmussen U, Rasmussen AK (2010) Autoimmunity in differentiated thyroid cancer: significance and related clinical problems. Hormones (Athens). 9: 109-117.

**100.** McLeod DS, Watters KF, Carpenter AD, Ladenson PW, Cooper DS, et al. (2012) Thyrotropin and thyroid cancer diagnosis: a systematic review and dose-response meta-analysis. J Clin Endocrinol Metab. 97: 2682-2692.

**101.** Chu X, Pan CM, Zhao SX, Liang J, Gao GQ, et al. (2011) A genome-wide association study identifies two new risk loci for Graves' disease. Nat Genet 43: 897-901.

**102.** Gudmundsson J, Sulem P, Gudbjartsson DF, Jonasson JG, Sigurdsson A, et al. (2009) Common variants on 9q22.33 and 14q13.3 predispose to thyroid cancer in European populations. Nat Genet 41: 460-464.

**103.** Hwang SJ, Yang Q, Meigs JB, Pearce EN, Fox CS (2007) A genome-wide association for kidney function and endocrine-related traits in the NHLBI's Framingham Heart Study. BMC Med Genet 8 Suppl 1: S10.

**104.** Nakabayashi K, Tajima A, Yamamoto K, Takahashi A, Hata K, et al. (2011) Identification of independent risk loci for Graves' disease within the MHC in the Japanese population. J Hum Genet 56: 772-778.

**105.** Panicker V, Wilson SG, Walsh JP, Richards JB, Brown SJ, et al. (2010) A locus on chromosome 1p36 is associated with thyrotropin and thyroid function as identified by genome-wide association study. Am J Hum Genet 87: 430-435.

**106.** Rawal R, Teumer A, Volzke H, Wallaschofski H, Ittermann T, et al. (2012) Meta-analysis of two genome-wide association studies identifies four genetic loci associated with thyroid function. Hum Mol Genet 21: 3275-3282.

**107.**  Zhao SX, Xue LQ, Liu W, Gu ZH, Pan CM, et al. (2013) Robust evidence for five new Graves' disease risk loci from a staged genome-wide association analysis. Hum Mol Genet 22: 3347-3362.

**108.** Ward LD, Kellis M (2012) HaploReg: a resource for exploring chromatin states, conservation, and regulatory motif alterations within sets of genetically linked variants. Nucleic Acids Res 40: D930-934.

**109.** [Raychaudhuri S, Plenge RM, Rossin EJ, Ng ACY, International Schizophrenia Consortium, et al. (2009) Identifying Relationships Among Genomic Disease Regions: Predicting Genes at Pathogenic SNP Associations and Rare Deletions. PLoS Genet 5:e1000534.](http://www.plosgenetics.org/doi/pgen.1000534)

**[110.](http://www.plosgenetics.org/doi/pgen.1000534)** [Cao S, Liu J, Chesi M, Bergsagel PL, Ho IC, et al. (2002) Differential Regulation of IL-12 and IL-10 Gene Expression in Macrophages by the Basic Leucine Zipper Transcription Factor c-Maf Fibrosarcoma. J Immunol 169: 5715-5725.](http://www.plosgenetics.org/doi/pgen.1000534)

**[111.](http://www.plosgenetics.org/doi/pgen.1000534)** [Zhang M, Clausell A, Robinson T, Yin J, Chen E, et al. (2012) Host Factor Transcriptional Regulation Contributes to Preferential Expression of HIV Type 1 in IL-4-Producing CD4 T Cells. J Immunol 189: 2746-2757.](http://www.plosgenetics.org/doi/pgen.1000534)
